# The role of personality, authoritarianism and cognition in the United Kingdom’s 2016 referendum on European Union membership

**DOI:** 10.3389/fpsyg.2023.1077354

**Published:** 2023-03-22

**Authors:** Chris Sumner, John E. Scofield, Erin M. Buchanan, Mimi-Rose Evans, Matthew Shearing

**Affiliations:** ^1^The Online Privacy Foundation, London, United Kingdom; ^2^Department of Psychology, University of Missouri, Columbia, MO, United States; ^3^Department of Analytics, Harrisburg University of Science and Technology, Harrisburg, PA, United States; ^4^Independent Researcher, London, United Kingdom

**Keywords:** authoritarianism, Brexit, cognition, numeracy, personality

## Abstract

**Introduction:**

The results of the United Kingdom’s 2016 referendum on European Union (EU) membership have highlighted deep societal divides. In six studies, we examined the role of personality traits, cognition and cognitive biases in relation to referendum voters’ choices.

**Methods:**

A total of 11,225 participants completed questionnaires and controlled experiments, which assessed differences in personality traits, levels of authoritarianism, numeracy, thinking styles, and susceptibility to cognitive biases including ideologically motivated numeracy and reasoning, framing, and the Dunning-Kruger effect.

**Results:**

Participants expressing an intent to vote to leave the EU reported significantly higher levels of authoritarianism and conscientiousness, and lower levels of openness and neuroticism than voters expressing an intent to vote to remain in the EU. When compared with Remain voters, Leave voters displayed significantly lower levels of numeracy and appeared more reliant on impulsive System 1 thinking. In the experimental studies, voters on both sides were found to be susceptible to the cognitive biases tested, with a general trend for Leave voters to show more bias than Remain voters.

**Discussion:**

These results raise important questions regarding the use and framing of numerical and non-numerical data for public consumption.

## Introduction

The United Kingdom’s 2016 referendum on European Union (EU) membership was one of the most divisive democratic choices presented to the UK electorate in a generation, with polls running almost neck-and-neck between February 2016 and June 2016 (NatCen Social Research, 2016) and resulting in a narrow majority of 51.9% in favor of leaving the EU. The referendum campaign and the months following the result have highlighted deep societal, regional, and generational divides over opinions on integration with and membership of the EU, which are unsurprising as the British have had highly conflicting opinions since the onset of the project (Inglehart, 1970). In seeking to better understand these divisions, scholars and political commentators have focused largely on age, education and “the left behind” (Dorling, 2016; Goodwin and Heath, 2016). However, the role of differing personalities, cognitive abilities, and cognitive biases have been largely overlooked throughout pre- and post-referendum analysis.

There is a long history of research in exploring links between personality and political orientation, attitudes, and beliefs. Of this research, scholars have consistently identified relationships between political orientation and personality (Carney et al., 2008; Sibley et al., 2012) and authoritarianism (Altemeyer, 1981; Jost et al., 2003; Hetherington and Weiler, 2009). It is only in relatively recent years that we have witnessed the emergence of studies investigating the role of personality in relation to voters’ attitudes to the EU, European identity and Euroscepticism (Schoen and Schumann, 2007; Hooghe and Marks, 2009; Tillman, 2015; Bakker and de Vreese, 2016).

In contrast to personality traits, the roles of numeracy, thinking styles and cognitive biases in relation to political attitudes have seen comparatively less research. However, scholars have observed how these factors appear to differ between conservatives and liberals (Iyer et al., 2012; Pennycook et al., 2012; Deppe et al., 2015; Yilmaz and Saribay, 2016, 2017). Given the volume and velocity of statistics used throughout the referendum campaign, some of which were misinterpretable and arguably misleading (Nakatudde, 2017), coupled with the tone of campaign literature and press coverage, the role of cognitive abilities, thinking styles and biases are an important consideration. Of particular interest are cognitive abilities such as numeracy (Peters et al., 2006), differing cognitive processes such as thinking styles (Stanovich and West, 2000) and susceptibility to cognitive biases such as the Dunning-Kruger effect (Kruger and Dunning, 1999) and the framing-effect (Kahneman and Tversky, 1979). Existing research lacks investigation into the link between cognitive ability, thinking styles, and biases in relation to attitudes to EU membership.

The contribution of the present studies is that of replicating and extending past work by examining personality traits, authoritarianism, numeracy and thinking styles in relation to UK EU referendum voters, and additionally examining the extent to which voters could potentially be influenced through cognitive biases. At the time of writing, there remains very little research into the role of personality and attitudes to the EU and of the research available, none had examined personality in relation to the binary choice of Leave (the EU) or Remain (in the EU), which was presented to UK voters, and none had examined the potentially confounding effects of age and sex. While there has been some research into the role of cognitive ability and biases in relation to political orientation, at the time of writing, there had been none in relation to attitudes to the EU. The combination of these factors is important given a growing need to understand the extent to which voters could be targeted and subsequently influenced through both traditional and social media. The present studies therefore address an important gap in understanding less commonly investigated factors and their influences on attitudes to EU membership among referendum voters.

### The big five model of personality

The relationships between personality and political orientation have been studied since at least the 1930s (Rentfrow et al., 2009) with studies exploring differences between liberals and conservatives accounting for a significant portion of the research (Carney et al., 2008). This research has predominantly focused on social liberalism and social conservatism. More recently, scholars have begun investigating relationships between personality and a number of different attitudes to the EU (Schoen and Schumann, 2007; Bakker and de Vreese, 2016; Curtis, 2016; Nielsen, 2016).

Political orientation is an important understudied consideration in the context of the present studies as the impetus for the referendum stemmed from a desire to reduce the growing threat of the anti-EU UK Independence Party (UKIP; Matthijs, 2013), coupled with deep historic divisions over membership of the EU within the Conservative party (Garry, 1995; Hooghe and Marks, 2009). Research has shown that, in the case of mainstream parties in the UK, there is a significant negative relationship between social conservatism and support for EU integration (Hooghe et al., 2004; Prosser, 2016). Mainstream parties are defined as the electorally dominant parties in the UK and include the Conservative Party, Labour Party, Liberal Democrats, Scottish National Party, and Plaid Cymru. To explain the rising support of right-wing populism in the UK, Ford and Goodwin (2014) note that UKIP’s appeal stems from three motives: a hard brand of Euroscepticism, a strong opposition to immigration and a dissatisfaction with the established political parties. It is important to note that Euroscepticism is not unique to right-wing and right-wing populist parties; an inverted U-Curve has been observed, in which both radical right and radical left parties attract Eurosceptic voters (Hooghe et al., 2004; Halikiopoulou et al., 2012). Given the consistency of extant research into political orientation and Euroscepticism, it is likely that the personality traits which attract people to right-wing mainstream and both radical right and radical left-wing political parties will be reflected in the cohort intending to vote Leave in the UK’s 2016 referendum on EU membership.

With regard to personality research, both in general and in the context of political orientation, the Five Factor Model (FFM), or the “Big Five,” has emerged as the most widely used empirical taxonomy of personality traits (Goldberg, 1992; John and Srivastava, 1999; McCrae and Costa, 1999). The Big Five model consists of five broad personality traits, namely Openness, Conscientiousness, Extraversion, Agreeableness and Neuroticism (Goldberg, 1992; John and Srivastava, 1999). General information regarding the Big Five can be found in the supplemental materials.^1^ Openness, conscientiousness and to a lesser extent, neuroticism have repeatedly been shown to correlate with people’s political orientation (Carney et al., 2008; Vecchione et al., 2011; Sibley et al., 2012). Extant research has shown that greater openness correlates with more liberal political beliefs, while greater conscientiousness correlates with more conservative political beliefs (McCrae, 1996; Jost et al., 2003; Ekehammar et al., 2004; Carney et al., 2008). While a smaller effect size, extant research has found that lower neuroticism correlates with more conservative political beliefs (Sibley et al., 2012). Relationships between political orientation and both agreeableness and extraversion appear less frequently in research, although agreeableness has been observed to have a negative but weak correlation with conservatism. The strength of the relationship between Agreeableness and political orientation may, in-part, be explained by conflicting lower-level traits. Specifically, the facet of compassion has been associated with liberalism, while politeness has been associated with conservatism (Hirsh et al., 2010). Given the negative relationship between social conservatism and support for EU integration (Hooghe et al., 2004; Prosser, 2016), a reasonable hypothesis is that Leave voters will, as a population, display higher conscientiousness together with lower openness, neuroticism and agreeableness than Remain voters.

Within the last decade, a small number of studies have begun to explore how personality traits correlate with attitudes to EU membership, identity, Euroscepticism and immigration (Schoen and Schumann, 2007; Gallego and Pardos-Prado, 2014; Bakker and de Vreese, 2016; Curtis, 2016; Nielsen, 2016). As with political orientation, both openness and conscientiousness appear to play a significant role in attitudes to the EU and Euroscepticism. Openness has been found to be positively correlated with support for the Euro currency, European Government, expansion of the EU and identification with Europe (Schoen and Schumann, 2007; Bakker and de Vreese, 2016; Curtis, 2016) possibly as a result of increased exposure to different cultures (Mondak, 2010). A negative relationship has been observed between conscientiousness and support for the EU and related topics (Schoen and Schumann, 2007; Kappe, 2015). Neuroticism has been found to be positively correlated with positive attitudes towards the EU, specifically in support of both widening and deepening the EU (Bakker and de Vreese, 2016). As with political orientation, there is far less consistency and strength in observed relationships between agreeableness in relation to attitudes to the EU. An example of this inconsistency is that agreeableness has been found to be negatively correlated with identification with Europe and deepening relationships with the EU, but also positively correlated with support for widening the EU and support for European Government (Schoen, 2007). The final Big Five trait, extraversion, has been observed to be negatively correlated with support for widening the EU, although this finding is limited to one paper (Bakker and de Vreese, 2016).

The findings from these nascent studies, coupled with the findings of papers exploring personality and political orientation suggest that Leave voters will, as a population, display higher conscientiousness and extraversion together with lower openness, neuroticism, and agreeableness. What is less well understood is the impact of age and sex on the effect of personality in relation to attitudes to EU membership, especially as different age cohorts may show different predispositions given their life experiences. Given the generational divides observed in the voting patterns of the referendum and that personality has been shown to change with age and differ between the sexes (Neyer and Asendorpf, 2001; Soto et al., 2011), this is an important factor to take into account.

### Authoritarianism

Given the relationship between social conservatism and Euroscepticism, it is important to examine other associated factors. In the present study, we examine authoritarianism, the study of which originated in the years following World War II, stemming from an effort to understand the rise of fascism in Europe during the 1930s and 1940s (Adorno et al., 1950). Although there is debate on whether authoritarianism is synonymous with conservatism (Crowson et al., 2005), studies repeatedly demonstrate a significant positive relationship between the two (Altemeyer, 1996; Duckitt and Bizumic, 2013). Further, and of importance, studies have found that people with higher levels of authoritarianism hold fundamentally different worldviews than people with lower levels; additionally, these differences have played a central role in structuring mass preferences and issue agendas in American politics (Hetherington and Weiler, 2009) and may help explain why attitudes to the EU cut across historic political party lines. Specifically, and in the context of American politics, research is indicating a better alignment between party affiliation and ideology (Fiorina and Levendusky, 2006); it is possible that this sorting is also an important aspect in the EU referendum vote. At the time of writing, two studies have explored the relationship between authoritarianism and Euroscepticism (Tillman, 2013; Kappe, 2015).

Authoritarianism, is said to be observable in early childhood, and has been described as a narrowly defined trait which may conceptually fall under Big Five traits and other facets of personality (Ekehammar et al., 2004). Authoritarians have been characterized by (a) “a high degree of submission to the authorities who are perceived to be established and legitimate”; (b) “a general aggressiveness, directed against various persons, which is perceived to be sanctioned by established authorities”; and (c) “a high degree of adherence to the social conventions which are perceived to be endorsed by society” (Altemeyer, 1981, p: 148). The Right Wing Authoritarian (RWA) scale (Altemeyer, 1981) is one of the more widely used scales for measuring authoritarianism in individuals, with scores on the RWA scale having been shown to “predict a broad range of attitudes and behaviors related to social, economic, and political conservatism as defined in the general culture at the time” (Jost et al., 2003, p: 345).

Despite its long history in political psychology, authoritarianism has only recently been directly researched in relation to EU attitudes where authoritarians were found to be more likely to express fears about the EU, oppose immigration and express pride in their nationality; they were also less likely to identify as European (Tillman, 2013; Kappe, 2015). Supporting these findings, a study using data from the 2015 British Election Study (Fieldhouse et al., 2015), found that authoritarian values were strongly associated with more negative views on immigration and minorities, and ultimately with support for UKIP (Kappe, 2015). Prior to these studies, research had observed relationships between EU attitudes and factors often associated with authoritarianism. For example, it has been observed that “Essentially, people are hostile toward the European project in great part because of their perceptions of threats posed by other cultures” (McLaren, 2002, p: 551). The importance of examining the role of authoritarianism in understanding support for the EU was underlined in a study which found that attitudes towards European integration had shifted from being primarily dominated by economic left–right aspects in 1958, to being primarily dominated by social liberal-conservative aspects in 2008 (Prosser, 2016). The social liberal-conservative spectrum is closely related to the authoritarian spectrum and appears to be playing an increasingly important role, indeed Prosser (2016, p: 16) further comments that “This finding aligns with much recent research on the role of factors such as hostility to immigration and authoritarianism, which fit within the conservative side of the social dimension, play in structuring citizen hostility towards European integration.”

Collectively, the evidence suggests that authoritarianism plays a significant role in attitudes to EU membership and therefore we expect a significant positive correlation between authoritarianism and a vote to leave the EU. Indeed, the ‘Vote Leave’ campaign message of ‘Take Back Control’ would seem well constructed to appeal to voters with more authoritarian attitudes as it may resonate with associated motives such as an intolerance for uncertainty and the need for structure and order (Jost et al., 2003; Hetherington and Weiler, 2009). Further, authoritarianism appears higher in older cohorts, with theories suggesting that older people grew up in times when authoritarian attitudes were more common (Ruffman et al., 2016). As such, we additionally expect to see higher scores for RWA and thus more support for a Leave vote among older generations.

### Numeracy, thinking styles and cognitive biases

In light of the widespread use of numerical claims and counterclaims throughout the referendum, many of which were arguably misinterpretable or misleading (Nakatudde, 2017), it is important to explore the role of numeracy and thinking styles in order to understand whether Leave and Remain voters consume and process information differently. Furthermore, considering the often rancorous nature of referendum arguments, in which many of the statistical claims were selectively reported (Nakatudde, 2017) and presented in tit-for-tat exchanges between rival referendum camps (Cushion and Lewis, 2017), it is additionally important to explore the roles of cognitive biases such as ideologically motivated reasoning, ideologically motivated numeracy, framing, and the Dunning-Kruger effect. In particular, it would be useful to understand if one set of voters were more susceptible to these biases than the other.

Numeracy, thinking styles, and cognitive biases have previously been examined in relationship to US political orientation (Kahan, 2013; Kahan et al., 2017), but at the time of writing, no studies had been identified which have examined whether there is a relationship between these factors and attitudes to EU membership and the EU in general. Evidence that voters on both sides of the referendum may have been susceptible to manipulation through an appeal to cognitive biases stems from a wide range of separate but related bodies of research. For example, research has shown that less numerate individuals are more susceptible to the way in which numerical information is framed (Peters et al., 2006) while other research has highlighted the effects of persuasion on voters (Jungherr et al., 2017). In order to better understand the extent to which it may be possible to target and influence both Leave and Remain voters, in addition to personality and authoritarianism, it appears important therefore to also explore the role of numeracy, thinking styles, and cognitive biases.

Any susceptibility to biases may be further influenced by other factors which could limit voters’ ability to make informed voting decisions. Two may be particularly important. Firstly, of the 28 EU member states, UK voters were found to be among the least knowledgeable about the EU (Eurobarometer, 2015). Secondly, theories such as ‘rational ignorance’ suggest that in general, “it is irrational to be politically well-informed because the low returns from data simply do not justify their cost in time and other scarce resources.” (Downs, 1957, p: 259).

Closely related to rational ignorance is the theory of rational irrationality (Caplan, 2001). Rational irrationality suggests that there is a relationship between the personal cost of a decision and the degree of rationality applied to making the choice. People are said to adjust the intellectual rigor they apply to a decision commensurate with the practical importance of the choice presented to them. That is, a person becomes increasingly likely to base decisions on previously held beliefs (the “bliss belief”) and less likely to apply intellectual rigor as the personal cost or impact associated with a choice decrease. The theories of rational ignorance and rational irrationality illustrate the importance of gaining a deeper understanding of the way in which referendum voters’ process information.

The importance of understanding the role of numeracy, thinking styles and biases was underlined in the run-up to the referendum when the British newspaper, the Daily Telegraph, reported that Professor Daniel Kahneman had warned that “voters are succumbing to impulsive gut feelings and irrational reflexes in the Brexit campaign with little regard for the enormous consequences down the road” (Evans-Pritchard, 2016). The present paper focuses on numeracy, thinking styles and biases in order to explore cognitive processing differences and similarities between voters; the following sections introduce each of these topics, respectively.

#### Numeracy

Numeracy refers to the ability to accurately interpret mathematical operations (Peters et al., 2006) and has been used in research for over a decade to measure skills such as financial and health care decision making (Reyna et al., 2009; Lusardi, 2012). Studies have found that many people lack basic numerical skills (Public Accounts Committee, 2008; Reyna et al., 2009; Kerr, 2010). Even in educated samples, a sizable proportion were likely to have difficulty with relatively simple numeracy problems (Lipkus et al., 2001). In the UK specifically, surveys of people aged 16–65 found that the number of respondents being classified at Level 2 or above in numeracy had decreased slightly from 25.5% in 2003 to 21.8% in 2011 (Harding et al., 2012). Level 2 is the equivalent to a GCSE pass grade, an exam typically sat at age 16. Moreover, the UK was the second worst nation in numeracy when compared with 23 other nations in the 2013 OECD Survey of Adult Skills (Kuczera et al., 2016). Taking into account our hypothesis that Leave voters will tend to be more authoritarian than Remain voters, and considering the relationships identified in previous research between intelligence and RWA (Deary et al., 2008; Heaven et al., 2011), conservatism, openness, and conscientiousness (Stankov, 2009; Hodson and Busseri, 2012), we expect to see lower numeracy in Leave voters as a population. Consideration was given to exploring intelligence rather than numerical risk literacy. While intelligence could be a potential moderator, we did not find an intelligence instrument that we could use, at no additional cost, within our web-based experiments. We therefore elected to examine numerical risk literacy.

#### Thinking styles

Thinking styles are the cognitive processes which individuals use to perceive, process and analyze information or problems presented to them. In this study, we focus on dual process theory and deductive reasoning. With regard to dual process theory, scholars have identified two distinct types of cognitive reasoning processes or thinking styles, which affect a person’s ability to interpret information. The first relates to low effort, impulsive and unconscious reasoning. The second relates to higher effort, conscious and analytical reasoning (Epstein, 1994; Sloman, 1996; Chaiken and Trope, 1999; Kahneman and Frederick, 2002; Kahneman, 2011). These are labeled as System 1 and System 2 processes, respectively, (Stanovich and West, 2000). The three-question Cognitive Reflection Test (CRT; Frederick, 2005) has emerged as the most widely used test for measuring a person’s tendency to override impulsive System 1 thinking, and to engage reflective System 2.

The second thinking style we examine is deductive reasoning. This refers to the process of applying an accepted rule to a specific statement in order to determine whether the statement is logically correct or not. One of the more widely known tests for examining deductive reasoning ability is Wason’s abstract 4-Card Selection task (Wason, 1966). Subsequent research has additionally shown that people tend to perform better at deductive reasoning tasks when the tasks involve realistic statements rather than abstract statements (Griggs and Cox, 1982).

Dual process theory and deductive reasoning are important considerations with regard to the referendum for broadly the same reasons as numeracy. That is, given the quantity of numerical and non-numerical data published about the EU and associated topics, the ability to accurately evaluate the data is of high importance in making informed decisions. Referendum campaigns and accompanying analysis not only featured numerical data, but a high velocity of numerical data covering a range of topics over a number of months, often in the form of tit-for-tat exchanges (Cushion and Lewis, 2017). It could be argued that voters were bombarded with an overwhelming and bewildering amount of numerical information.

With regard to the relationship between cognitive reflection and political orientation, there is a little more published research, of which studies have found that social conservatives tend to be less reflective (Deppe et al., 2015). This finding was theorized as conservatives relying on heuristics associated with implicit reasoning (Iyer et al., 2012; Pennycook et al., 2012; Deppe et al., 2015). Given the positive relationship identified in previous research between cognitive reflection, liberalism, and actively open-minded thinking (Campitelli and Labollita, 2010; Haran et al., 2013), we expect to see Remain voters to perform slightly better at the CRT than Leave voters.

Considering the extant research evidence which suggests that Remain voters ought to possess greater numerical risk literacy than Leave voters, we may also expect Remain voters to perform better than Leave voters at the selection task. However, considering the characteristics of rule adherence associated with social conservatism, we anticipate that any performance difference may be reduced or eliminated when the selection task is in the form of a concrete social rule. Overall, given the relative absence of research into deductive reasoning, personality and political orientation, this hypothesis remains highly speculative.

#### Cognitive biases

The final body of research we explore examines whether susceptibility to cognitive biases differs between voters. Of these biases, we focus on ideologically motivated reasoning and numeracy (Kahan, 2013; Kahan et al., 2017), overconfidence (Kruger and Dunning, 1999) and the tone or framing of information (Kahneman and Tversky, 1979). While research into numeracy and thinking styles provides evidence that liberals tend to have a greater ability in correctly interpreting numbers (Stankov, 2009; Hodson and Busseri, 2012), scholars have repeatedly found evidence that motivated reasoning (Kunda, 1990; Gollust et al., 2009) and ideologically motivated reasoning (Cohen, 2003; Gollust et al., 2009; Kahan, 2013) affects how people process information. Ideologically motivated numeracy (Kahan et al., 2017), for instance, can significantly reduce a person’s ability to interpret a set of numbers, if those numbers do not support pre-existing beliefs.

In a study dubbed ‘The Most Depressing Brain Finding Ever’ (Kaplan, 2013), Kahan et al. (2017) conducted an experiment where participants were given a set of numbers to interpret. One group of participants was asked to interpret numbers about a skincare product and state whether the cream reduced rashes. Regardless of political orientation, participants fared similarly at this task. Another group of participants were asked to interpret numbers about handguns and whether carrying them increased or decreased crime. Kahan et al. found that when the numbers conflicted with participants’ pre-existing ideological beliefs on gun control, their ability to correctly interpret the numbers was greatly diminished. Further, they found that participants with greater ability to suppress System 1 thinking fared worse, being affected more by their ideological views. Kahan’s and other’s findings related to ideologically motivated numeracy and reasoning suggest that voters on both sides of the debate are likely to succumb to this bias. Further, assuming that the hypothesis that Remain voters have greater numeracy ability and are more likely to use System 2 thinking, it is plausible that they will be affected by this bias to a greater extent than Leave voters.

The second bias we explore is the Dunning-Kruger effect, a bias where people with lower abilities to perform a task tend to overestimate their performance at that task. Conversely, the greater someone’s ability, the less they overestimate their performance (Kruger and Dunning, 1999). In particular, instead of performance, we focus on the bias of over or underconfidence between Leave and Remain voters. Given the complexity of issues surrounding EU membership, the multifaceted arguments for remaining in it or leaving it, and the generally low levels of knowledge voters possessed about the EU prior to the referendum (Eurobarometer, 2015), it is difficult to imagine voters having sufficient knowledge or time to make a fully informed decision. This effect is likely to apply to voters on both sides of the debate and may be further exacerbated by the effects related to the theories of rational ignorance (Downs, 1957) and rational irrationality (Caplan, 2001). Moreover, when rational ignorance and rational irrationality meets the Dunning-Kruger effect, it is entirely possible that a majority of voters may have believed that they understood more about the UK’s membership of the EU and how it benefits or hinders the UK better than they actually did. However, recent research, published after the original planning and draft of this manuscript, has shown that the Dunning-Kruger effect may be a statistical artifact and sometimes does not replicate (Gignac and Zajenkowski, 2020; Hofer et al., 2022). Given the lack of clear direction in which group may “perform” better, we used over and underconfidence to examine the level of bias in the voters.

The final bias we explore is framing, that is, examining how voters react depending on how the same information is presented. During the EU referendum campaign, the Stronger in Europe campaign was accused of focusing on the negative consequences of the UK leaving the EU and was labeled as ‘Project Fear’ (Johnson, 2016). This campaign raises an important consideration, that is, were voters susceptible to the way in which a message was pitched, or framed? Perhaps the most famous experiment to examine framing is Tversky and Kahneman (1981) Asian disease problem. The experiment found a reversal in an individual’s choices depending on the way in which the choices were framed. In the experiment, when the choices were presented in terms of lives saved, participants tended to prefer a safer option (guaranteed to save 200 out of 600 people versus a riskier choice with a 2/3 probability that 600 people would die). However, when the choices were presented in terms of expected deaths (guaranteed that 400 people would die versus 2/3 probability that 600 people will die), participants tended to choose the riskier option. The debates on the UK membership of the EU have arguably spanned decades, providing ample opportunity for campaigners and the media to knowingly or unknowingly use framing to shape public opinion. Research has shown that if EU enlargement was presented as a risk, people’s support was generally lower than if it was presented as an opportunity (Schuck and de Vreese, 2006). The study also found that less politically knowledgeable individuals were more affected by experimental manipulation and more susceptible to risk framing. On balance, and in relation to susceptibility to framing, we do not therefore anticipate any significant differences between Leave and Remain voters.

Overall, based on available research, we hypothesize that Remain voters will exhibit better numeracy, possess a greater ability to suppress System 1 thinking and perform better at abstract reasoning than Leave voters. We hypothesize that it is likely that voters on both sides will perform similarly when the reasoning task is in the form of a social rule and be susceptible to cognitive biases in more-or-less, equal measures. Consideration has been given to research which suggests that political extremists appear to be less affected by biases (Brandt et al., 2015), although, on balance, extreme political views should be relatively equally distributed on both Leave and Remain sides.

### The present studies

The aim of the present studies was to investigate personality, authoritarianism, and cognitive differences in voters who intended to vote Leave as compared to those who intended to vote Remain. The studies replicate and extend prior research, but in the context of voters in the UK’s referendum on EU membership. Prior research suggests that we should expect differences in personality and cognitive skills, but that susceptibility to cognitive biases such as framing and the Dunning-Kruger effect, should apply to voters across the electorate. Gaining a deeper understanding of the differences and similarities between Leave and Remain voters is an important area of study, not only to better understand UK society, but also to contribute to research exploring the efficacy of psychographic targeting. In light of allegations of psychographic targeting during the referendum (Shipman, 2017), it is important to understand whether, and to what extent, knowledge of voters’ core psychological characteristics and biases could be exploited to influence the way they form early opinions and subsequently process information. The following hypotheses were therefore examined:

**Personality**. We primarily expect openness, agreeableness, and neuroticism to be positively related to a vote to Remain (i.e., higher scores) and conscientiousness and extraversion to be negatively related to a vote to Remain (i.e., lower scores). In line with extant research, we expect openness and conscientiousness to play the largest roles.

**Authoritarianism**. We expect Leave voters to score significantly higher in authoritarianism.

**Numeracy**. We expect Remain voters to perform better in relation to numeracy.

**Thinking styles**. Using the three question Cognitive Reflection Test as a measure of thinking styles, we anticipate that Remain voters will have a greater ability to override System 1 thinking, thereby scoring higher in the test. Using the Wason Card Selection task, we expect Remain voters to outperform Leave voters at abstract reasoning, but that both Leave and Remain voters will perform similarly when the reasoning task is in the form of a social rule.

**Biases**. First, ideologically motivated reasoning was measured using the experimental component from Kahan (2013), and we do not expect to see significant differences between Leave and Remain voters. However, when examining Kahan et al. (2017), we expect Remain voters to perform better at the control question, but to lose any advantage when the question is contrary to their assumed beliefs and be more affected by ideologically motivated numeracy than Leave voters. Second, using the Asian disease and Sure gain/Sure loss problems (Tversky and Kahneman, 1981) as experimental components, we do not expect to see significant differences between Leave and Remain voters in relation to framing. Last, we expect voters on both sides to be equally susceptible to the Dunning-Kruger over and underconfidence bias, but older voters to fare worse than younger voters because of the research showing age effects on heuristics (Besedeš et al., 2012). As noted in the introduction, the Dunning-Kruger effect may not replicate or be statistically reliable (Gignac and Zajenkowski, 2020; Hofer et al., 2022); however, in order to avoid HARKING, we have included our original hypothesis and interpretation in this manuscript (Kerr, 1998).

No hypotheses were pre-registered, but all hypotheses were derived before data collection and analysis.

## Methods

### Participants

Overall, six studies were completed by recruiting participants through Facebook advertising targeted at users over the age of 18 and living in the United Kingdom. In sum, and split across the six studies, 11,225 participants were recruited with 6,866 males and 4,359 females taking part. Age groups were constructed into 18–34 (4,130), 35–54 (3,550), and 55+ (3,545). Participants received instant results regarding their scores but were not compensated for their participation. A complete breakdown of sex and age groups by study can be found online (see footnote 1). All data was collected between the dates of 9th April 2016 and 23rd June 2016. Ethical approval was received from the Online Privacy Foundation Ethical Board in London, United Kingdom (2016/12–27/2).

### Materials and procedures

Purpose-built web-survey applications were constructed for each study in order to collect basic demographic information consisting of Sex (Male, Female), age group (18–34, 35–54 and 55+), voting intention (Leave, Remain), and responses to each instrument described below. For clarity, we will discuss the studies grouped by dependent variable, as these correspond to the data screening and analyses described below. All studies included demographic variables. Table 1 indicates the dependent variables presented in each study.

**Table 1:** Variables presented in each study.

	Study					
Variables	1	2	3	4	5	6
Personality traits	X					
Authoritarianism: RWA				X	X	
Thinking styles: Cognitive reflection task	X	X				X
Thinking styles: Wason card deductive reasoning			X			
Biases: Dunning-Kruger effect			X			
Biases: Berlin numeracy task and motivated reasoning		X				
Biases: Motivated numeracy						X
Biases: Framing – Asian disease				X	X	
Biases: Framing – sure gain / sure loss					X	

**Voting Intention**. Participants were asked to select one option from a dropdown list, as follows: If the referendum were today, how would you vote? with the options: *Leave the EU, Remain in the EU, Spoil the ballot paper, Not vote.* In the UK electoral system, spoilt ballot papers include those with votes for more than one candidate; those where anything is written or marked by which the voter can be identified except the printed number and other unique identifying marks on the back; and those which are unmarked or void.

**Personality Traits**. Participants’ Big Five personality traits were assessed using the 44-question Big Five Inventory (John et al., 2008), providing measures of Openness (alpha = 0.77), Conscientiousness (alpha = 0.82), Extraversion (alpha = 0.85), Agreeableness (alpha = 0.76), and Neuroticism (alpha = 0.84). Participants were asked to indicate their response to each question on a five-point Likert style scale ranging from *strongly disagree* (1) to *strongly agree* (5).

**Authoritarianism**. Participants’ authoritarianism was assessed using the 20-question Right Wing Authoritarianism scale (Altemeyer, 2007) in which they were asked to indicate their response to each question on a nine-point Likert style scale ranging from *very strongly disagree* (0) to *very strongly agree* (8). The alpha in this dataset was 0.88.

**Numeracy**. Participants’ numerical risk literacy was assessed using the four-question multiple choice format, Berlin Numeracy Test (Cokely et al., 2012). The four questions are designed to measure both numeracy and risk literacy in populations with moderate-to-highly numerate individuals.

#### Thinking styles

***Cognitive Reflection Test***. Participants’ thinking styles were assessed using the three question Cognitive Reflection Test (Frederick, 2005), which is designed to measure a person’s ability to suppress an intuitive and spontaneous (System 1) wrong answer in favor of a reflective and deliberative (System 2) right answer. The procedure followed is as described in da Silva et al. (2015), in which participants selected one of several options for each answer, instead of the free response method described in Frederick (2005). In order to better differentiate between System 1 and System 2 thinkers, results were scored as a 1 for a correct answer, −1 for choosing the intuitive but incorrect answer and a 0 for all other incorrect answers.

***Wason Card Selection***. Participants’ deductive reasoning ability was assessed using a version of Wason’s abstract 4-Card Selection task (Wason, 1966), together with the concrete social rule drinking age problem (Griggs and Cox, 1982). For each set of four cards shown, participants received a point (1) if they selected only the correct cards and left the remaining cards unselected.^2^

#### Biases

***Dunning-Kruger Effect**.* Participant’s susceptibility to the Dunning-Kruger bias was measured by asking participants how many of the Wason Card Selection reasoning problems they thought they had answered correctly and how they believed they had ranked, in quartiles, in comparison to other participants. Participants’ actual performance was then subtracted from their estimated performance to provide a numeric measure of the Dunning-Kruger effect (Kruger and Dunning, 1999; Dunning, 2011). Higher scores represent an overconfidence bias, regardless of performance, while lower scores represent an underconfidence bias.

***Ideologically Motivated Reasoning**.* Participants’ manifestations of ideologically motivated reasoning were measured by replicating the experimental component of Kahan (2013) research. The present study deviates slightly from Kahan’s, as it additionally includes the Berlin Numeracy Test. Participants were randomly assigned to one of three groups, ‘Control’, ‘Leave biased’ and ‘Remain biased’. As in Kahan’s experiment, all participants reported, using a six-point Likert style scale ranging from *strongly disagree* (1) to *strongly agree* (6) answers to “I think the word-problem test I just took supplies good evidence of how reflective and open-minded someone is.” These responses were used to measure participants’ test bias. Prior to answering this question, participants viewed a statement unique to the group they were assigned to. The control group received text stating that “psychologists believe the questions you have just answered measures how reflective and open-minded someone is.” The Leave biased group additionally received text stating that “in one recent study, a researcher found that Leave voters tend to get more answers correct than Remain voters” while the Remain biased group additionally received text stating that “in one recent study, a researcher found that Remain voters tend to get more answers correct than Leave voters.”

***Ideologically Motivated Numeracy**.* Participants’ manifestations of ideologically motivated numeracy were measured by replicating Kahan et al. (2017) and changing the context of the experimental questions from gun control to immigration ideology. Participants were randomly assigned to one of four groups: A, B, C or D. Groups A and B received a question on the same topic, a question concerning skin cream and its effect on rash. Groups C and D each received a question regarding immigration and its effects on crime. Participants were presented with a contingency table which they were asked to interpret in order to determine, in the case of the control questions, whether skin cream increased or decreased a rash, or, in the case of the experimental questions, whether immigration increased or decreased crime. The numbers in the contingency table remained the same for all four groups, only the outcomes were modified. Participants received a point (1) for a correct answer and a no points (0) for an incorrect answer. The exact questions shown to participants can be found online(see footnote 1).

***Framing***. Participant’s susceptibility to framing was measured using the ‘Asian disease problem’ (problem 1) and the ‘Sure gain/Sure loss’ problem (problem 3; Tversky and Kahneman, 1981). Participants were randomly assigned to either the positive or negative frame for each of the framing problems presented to them and then asked to indicate their preference for each of them. Participants received a point (1) if they selected the risky choice and received no points (0) if they selected the risk avoidant choice.

## Results and discussion

### Data screening and analysis

Data were first screened prior to all analyses presented in the current manuscript. Data screening included checking for accuracy, missing data, and outliers. Common statistical assumptions were also checked, including additivity, normality, linearity, and homogeneity. If outliers were identified in any dataset, those identified cases were removed from any further analyses for that section. Outliers were defined as *z*-scores or Mahalanobis distance scores that were *p* < 0.001 away from the mean score (i.e., z-scores greater than 3 and χ^2^ scores greater than the expected values for the number of variables in the analysis as degrees of freedom, Cohen et al., 2003). Table 2 shows each analysis and whether screening and statistical assumptions were met, at the multivariate level, using procedures from Tabachnick and Fidell (2012). When assumptions were violated, an appropriate correction was employed as discussed within each section below. The analyses below also focus mainly on effects concerning participants’ vote intention and any interactions with vote intention. Effects of age group and sex, while included in analyses, are presented in detail in the supplementary materials online (see footnote 1). Full summary statistics for all analyses are also accessible in supplementary materials. The large sample size for these studies was used to maximize power.

**Table 2:** Data screening across all analyses.

Assumption	Personality	RWA	RWA 2	Berlin numeracy & motivated reasoning	Motivated numeracy	CRT	Wason Card DK	Framing Asian Disease	Framing sure gain / sure loss
Outliers	4	36	18	0	0	0	0	0	0
Accuracy	Y	Y	Y	Y	Y	Y	Y	Y	Y
Missing Data	Y	Y	Y	Y	Y	Y	Y	Y	Y
Additivity	Y	Y	Y	Y	Y	Y	Y	Y	Y
Normality	Y	Y	Y	Y	Y	Y	Y	Y	Y
Linearity	Y	Y	Y	Y	Y	Y	Y	Y	Y
Homogeneity	N	N	N	Y	N	Y	N	N	N
Males	1,338	2,257	1,390	949	1,123	2,290	985	2,277	1,403
18–34	649	888	545	350	298	1,002	465	898	551
35–54	406	676	385	389	380	795	220	679	387
55+	283	693	460	210	445	493	300	700	465
Females	1,043	1,243	551	498	958	1,542	525	1,259	556
18–34	449	419	188	113	200	562	198	422	190
35–54	273	347	162	185	356	459	130	350	163
55+	321	477	201	200	402	521	197	487	203

RWA: right wing authoritarianism, RWA2: right wing authoritarianism second analysis, CRT: cognitive reflection task, DK: Dunning-Kruger.

### Personality

A 2 (Sex) × 3 (Age) × 2 (Vote intention) between-subjects MANOVA was first analyzed with sex (male, female), age group (18–34, 35–54, 55 and above), and referendum vote intention (Leave, Remain), with its effects on the Big Five personality factor scores. Table 2 includes *N* group values. Significant multivariate main effects were found for referendum vote intention, *F*(5, 2,365) = 26.06, *p* < 0.001, η_p_^2^ = 0.05. No interaction effects were found between sex or age with referendum vote intention. Therefore, the results below just investigate the main effect of referendum vote intention. There was a significant interaction between sex and age group, *F*(10, 4,730) = 2.38, *p* = 0.01, η_p_^2^ = 0.01, replicating prior research considering age and sex differences to be important considerations. Specifically, a number of studies have found that conscientiousness and agreeableness increase with age, while neuroticism decreases with age (Neyer and Asendorpf, 2001; Soto et al., 2011). A detailed description of these replicated findings is available online in the supplementary material.

**Figure 1:**
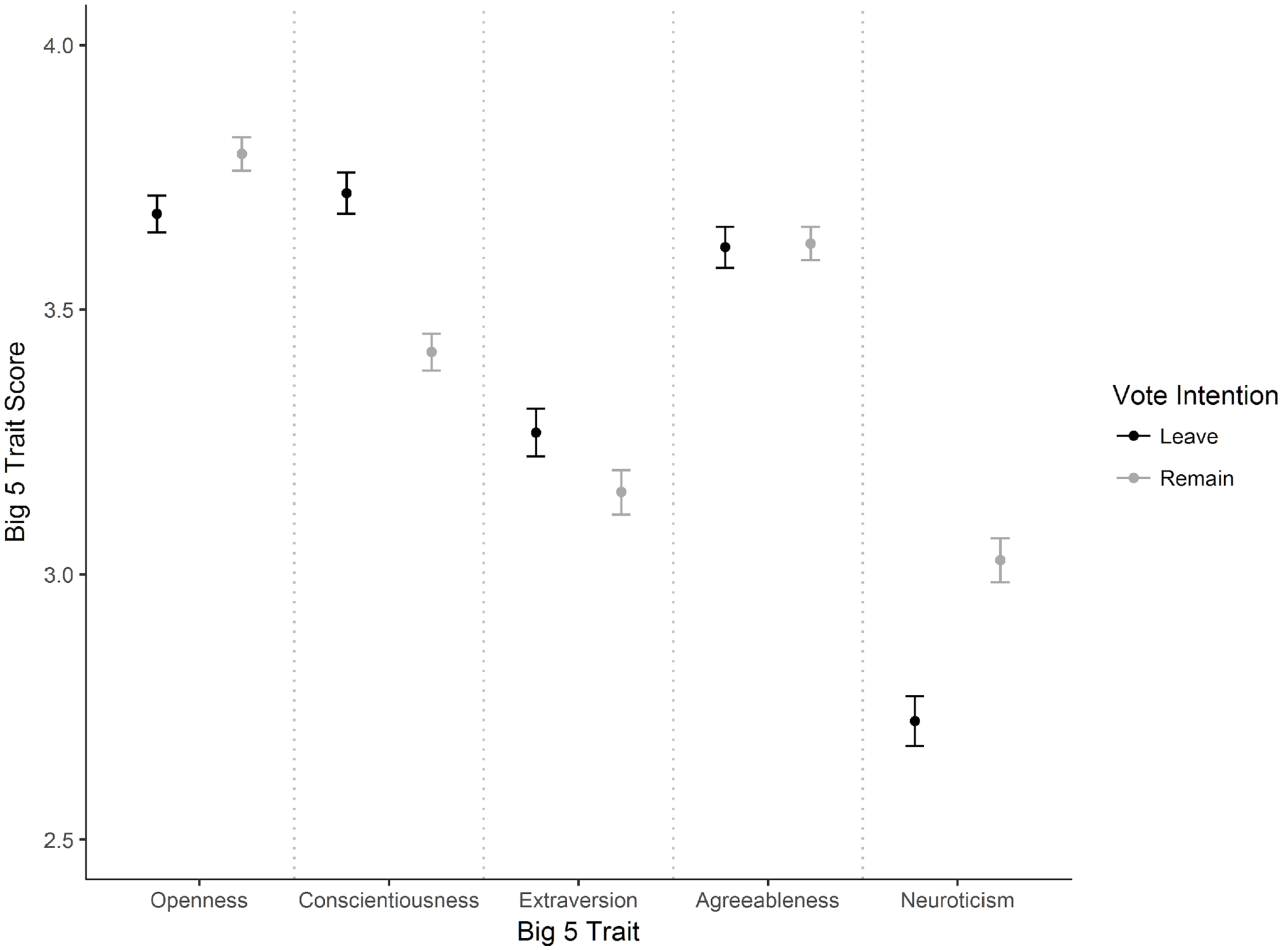
Vote intention by big five scores. Error bars represent 95% confidence intervals of the mean.

While investigating differences in specific dependent variables as a follow up to the MANOVA test, data indicated problems with univariate normality, skewness and kurtosis, and homogeneity in some dependent variables. To circumvent this problem, a series of Wilcoxon-Mann–Whitney rank sum tests were employed, while applying Bonferroni corrections to control for multiple comparisons. Figure 1 shows mean scores between the two types of EU referendum voters considering the five different personality traits and is described below in order of descending effect size. Leave voters (*M* = 3.72, *SD* = 0.63) had higher scores of conscientiousness than Remain voters (*M* = 3.42, *SD* = 0.66), *W* = 877,600, *p* < 0.001, *d* = 0.46. Considering neuroticism, Leave voters (*M* = 2.72, *SD* = 0.76) scored lower than Remain voters (*M* = 3.03, *SD* = 0.79), *W* = 5,539,900, *p* < 0.001, *d* = 0.39. Leave voters (*M* = 3.68, *SD* = 0.56) also scored lower in terms of openness than Remain voters (*M* = 3.79, *SD* = 0.60), *W* = 610,020, *p* < 0.001, *d* = 0.19. Leave voters (*M* = 3.27, *SD* = 0.73) had higher scores of extraversion than Remain voters (*M* = 3.15, *SD* = 0.78), *W* = 747,660, *p* < 0.001, *d* = 0.15. No significant differences were found between Leave voters (*M* = 3.62, *SD* = 0.63) and Remain voters (*M* = 3.62, *SD* = 0.60) in terms of agreeableness, *W* = 685,350, *p* = 0.75, *d* = 0.01.

For the most part, we found support for our hypotheses with Leave voters self-reporting higher scores in conscientiousness and extraversion than Remain voters and Remain voters self-reporting higher scores in neuroticism and openness than Leave voters. The exception which did not support our hypothesis, was that we did not observe any significant differences in agreeableness between Leave and Remain voters.

Our hypotheses were informed by a large body of research consistently showing the role the Big Five personality traits play in relation to both social conservatism (Carney et al., 2008; Vecchione et al., 2011; Sibley et al., 2012) and Euroscepticism (Schoen and Schumann, 2007; Gallego and Pardos-Prado, 2014; Bakker and de Vreese, 2016; Curtis, 2016; Nielsen, 2016), with openness, conscientiousness and neuroticism expected to play the largest roles. While there were many reasons for people voting Leave or Remain, our hypotheses were based on an assumption that the vote would encapsulate and correlate very strongly with overall levels of positive or negative perceptions of the EU as an institution. However, in reality, this would not be a perfect positive correlation. For example, for portions of the electorate, fears over the future of the economy could have taken precedence over their perceptions of the EU as an institution in terms of its democratic legitimacy, and its broader social and political impacts. While the results broadly support our hypotheses, extant research suggests that openness and conscientiousness (Bakker and de Vreese, 2016) should have been the dominant traits in determining an individual’s vote intention, but instead we found conscientiousness and neuroticism to have larger effects. Our hypothesis of Leave voters self-reporting higher levels of extraversion than Remain voters was also supported. Previous research has found extraversion to be associated with higher levels of participation in a broad range of political activities (Gerber et al., 2011). It is therefore possible that Leave voters were more politically invested and motivated than Remain voters. Future research could examine personality in relation to the strength of feeling voters held with regard to each of the key referendum issues.

Previous research has noted the interaction between neuroticism and openness (Andreasen, 2005), however, this work does not fully explain why neuroticism appeared to play the more dominant role in the differences between voters than existing research suggested. One possible explanation is that the significantly higher levels of neuroticism in younger voters and female voters, both of which were less likely to vote Leave, helped to inflate the overall effect size. A second possible explanation is that, in comparison to Leave voters, Remain voters were much more concerned by the uncertainty of leaving the EU, and of a change to the status quo. It is possible that this uncertainty was reflected in people with higher levels of neuroticism.

One trait, agreeableness did not support our hypothesis, but along with extraversion was also a trait with inconsistent research results in relation to attitudes to the EU. Previous research had noted positive correlations for widening the EU and for a European Government, but also a negative correlation for deepening the EU (Schoen, 2007; Bakker and de Vreese, 2016). Given these inconsistencies, it is perhaps unsurprising that agreeableness did not play a significant role in voter differences.

### Authoritarianism

Permutation tests (Good, 1994)^3^ (see footnote 1)were fitted to test 2 (Sex) × 3 (Age) × 2 (Vote intention) ANOVAs for the main effects of sex, age, and vote intention on RWA scores due to issues with homogeneity and univariate skew/kurtosis. There was a significant interaction between age and vote intention, *MSE* = 3,426, *p* < 0.001, iterations = 5,000. This interaction was broken down by analyzing differences between vote intention, split on the variable of age, with *post-hoc* permutation tests. For participants 18–34, Leave voters (*M* = 71.31, *SD* = 21.91) had higher RWA scores than Remain voters (*M* = 49.99, *SD* = 16.88), *p* < 0.001, *d* = 1.14. For participants 35–54, Leave voters (*M* = 72.67, *SD* = 21.95) had significantly higher RWA scores than Remain voters (*M* = 47.56, *SD* = 16.43), *p* < 0.001, *d* = 1.33. Considering participants 55 and older, Leave voters (*M* = 80.49, *SD* = 20.66) also had higher RWA scores than Remain voters (*M* = 53.55, *SD* = 20.29), *p* < 0.001, *d* = 1.31.

**Figure 2:**
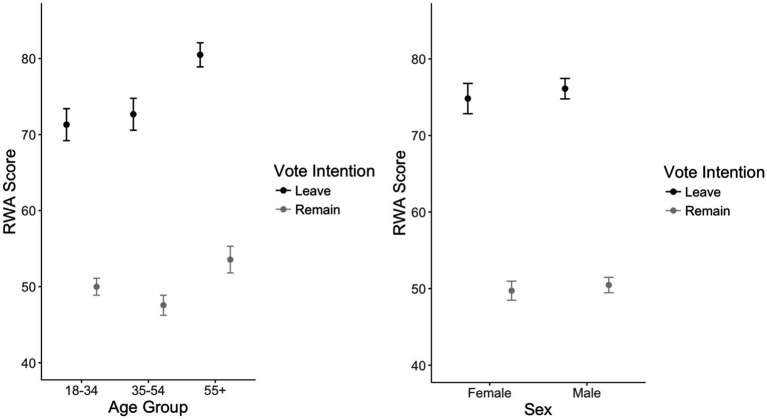
Differences in authoritarianism scores for leave and remain votes by age group (left) and by sex (right). Error bars represent 95% confidence intervals of the mean.

There was also a significant interaction between sex and vote intention, *MSE* = 2,674, *p* < 0.001, iterations = 5,000. For *post-hoc* analyses of this interaction, differences between vote intention were examined, split by the variable of sex. For females, Leave voters (*M* = 74.82, *SD* = 21.74) had higher RWA scores than Remain voters (*M* = 49.72, *SD* = 17.62), *p* < 0.001, *d* = 1.30. Considering males, Leave voters (*M* = 76.11, *SD* = 21.81) also scored significantly higher than Remain voters (*M* = 50.46, *SD* = 17.95), *p* < 0.001, *d* = 1.30. Figure 2 shows the interactions between both sex and vote intention, as well as age and vote intention. These results support our hypothesis, showing that Leave voters had higher RWA scores than Remain voters across all sexes and age groups.

Additionally, a second analysis on a subset of RWA scores was performed using the dependent variable of voter decision which included the option of “Undecided” alongside “Leave” and “Remain.” Permutation analyses were performed. A complete table of results is available online. The 2 (Sex) × 3 (Age) × 3 (Vote decision) ANOVA indicated an interaction for sex, age, and voter decision, *MSE* = 873, *p* < 0.001. The interaction was examined by splitting the sex variable and separate permutation ANOVAs were examined.

For male participants, the two-way interaction of age and voting was not significant, *MSE* = 850, *p* = 0.07; however, the main effect of vote was significant, *MSE* = 1,010,364, *p* < 0.001. Pairwise permutation tests were run for the main effect of voter decision. This result indicated that undecided voters (*M* = 62.89, *SD* = 22.25) scored directly in-between Leave (*M* = 77.35, *SD* = 21.46, *p* < 0.001, *d* = 0.53) and Remain voters (*M* = 47. 97, *SD* = 15.98, *p* < 0.001, *d* = 0.95). Leave voters also scored higher than Remain voters on the RWA scale, *p* < 0.001, *d* = 1.57.

**Figure 3:**
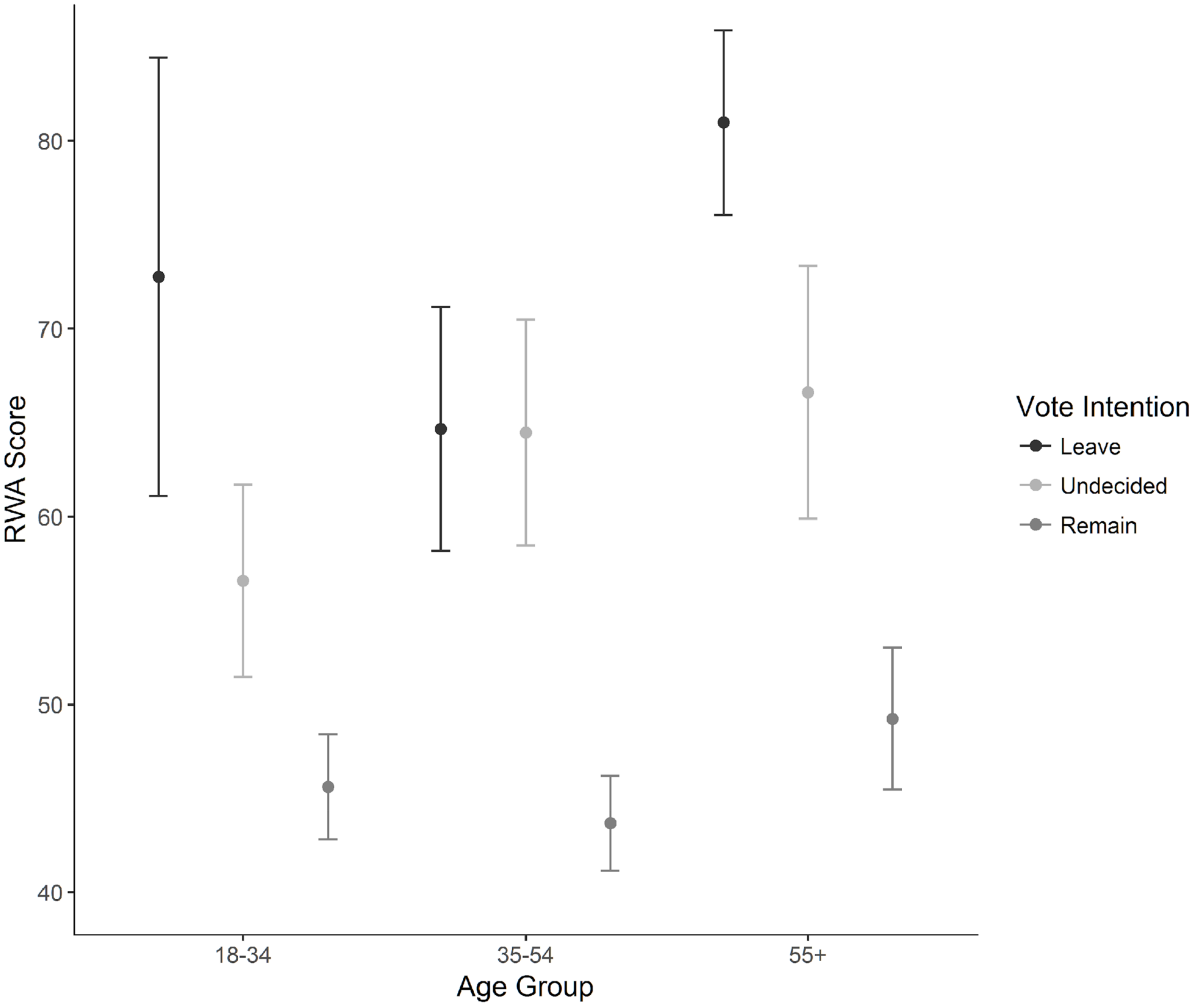
Differences in authoritarianism scores for females by age group. Error bars represent 95% confidence intervals of the mean.

For female participants, a two-way interaction of age and vote was significant, *MSE* = 1079.20, *p* < 0.05. Data were further split by age to examine this interaction among female participants. For young female voters, Leave voters (*M* = 72.76, *SD* = 22.71) had higher RWA scores than both Remain voters (*M* = 45.63, *SD* = 14.78, *p* < 0.001, *d* = 1.69) and undecided voters (*M* = 56.81, *SD* = 19.99, *p* < 0.001, *d* = 0.76). Young female undecided voters had higher RWA scores than Remain voters, *p* < 0.001, *d* = 0.65. For female voters 35–54, Remain voters (*M* = 43.69, *SD* = 11.39) had lower RWA scores than both Leave voters (*M* = 64.68, *SD* = 20.26, *p* < 0.001, *d* = 1.41) and undecided voters (*M* = 64.48, *SD* = 19.25, *p* < 0.001, *d* = 1.43). There were no differences between undecided and Leave voters, *p* = 0.96. For female participants 55 and over, Leave voters (*M* = 80.97, *SD* = 21.96) had higher RWA scores than both Remain voters (*M* = 49.25, *SD* = 15.94, *p* < 0.001, *d* = 1.64) and undecided voters (*M* = 66.62, *SD* = 23.94, *p* < 0.01, *d* = 0.62). Undecided voters also had higher RWA scores than Remain voters, *p* < 0.001, *d* = 0.88. Figure 3 shows average RWA scores for female participants grouped by voter decision, across the three age groups.

As with personality, our hypothesis was informed by a robust body of research repeatedly showing a significant relationship between authoritarianism, social conservatism (Altemeyer, 1996; Sibley and Duckitt, 2008) and the role of personality traits in the previous section. Furthermore, nascent research has identified significant correlations between authoritarianism and negativity towards the EU (Tillman, 2013; Kappe, 2015). Our results support these nascent studies, additionally observing how the difference in levels of authoritarianism between voters were noticeable and significant regardless of age group and sex.

Turning to the undecided voters, we find them caught between the higher authoritarian Leave voters and the lower authoritarian Remain voters. It is therefore possible that undecided voters were less likely to be instinctively drawn to one side of the debate or the other, making their referendum vote choice harder. Additionally, we found that 73% (*N* = 1,959) of voters had already decided on their vote decision by February 2016, the month the referendum was announced and 4 months prior to the vote.

Finally, it should be noted that the large effect sizes observed may be due to the use of the RWA scale. It has been argued that while the RWA scale is a reliable and empirically validated measure of authoritarian attitudes, it is not measuring authoritarian predisposition (Stenner, 2005). This may explain why, in comparison to the RWA, we observed lower effect sizes in the personality traits of openness and conscientiousness, the traits most commonly associated with authoritarianism. Future research should additionally consider measures such as the child rearing scale (Feldman and Stenner, 1997).

### Numeracy

A 2 (Sex) × 3 (Age) × 2 (Vote intention) between-subjects ANOVA was analyzed using the Berlin Numeracy Test scores as the dependent variable. There were no significant interactions between sex, age, or vote intention. There was a significant main effect of vote intention, *F*(1, 1,435) = 45.77, *p* < 0.001, η_p_^2^ = 0.03. Remain voters (*M* = 2.08, *SD* = 1.22) scored significantly higher than Leave voters (*M* = 1.53, *SD* = 1.09), lending support to the main hypothesis. We additionally found a significant main effect of sex, *F*(1, 1,435) = 29.91, *p* < 0.001, η_p_^2^ = 0.02, with male participants (*M* = 1.98, *SD* = 1.19) having significantly higher scores than female participants (*M* = 1.54, *SD* = 1.15). There was also a significant main effect of age, *F*(2, 1,435) = 11.70, *p* < 0.001, η_p_^2^ = 0.01. For *post-hoc* analysis, three independent samples *t-*tests with Bonferroni corrections were run. Participants 18–34 (*M* = 2.16, *SD* = 1.23) scored higher than both participants 35–54 (*M* = 1.82, *SD* = 1.16, *p* < 0.001, *d* = 0.29) and participants 55 and over (*M* = 1.47, *SD* = 1.08, *p* < 0.001, *d* = 0.59). Participants 35–54 also scored significantly higher than participants 55 and over, *p* < 0.001, *d* = 0.31.

Our findings from the Berlin Numeracy Test were consistent with our hypothesis that Remain voters would perform better than Leave voters. We established this hypothesis based on prior research into intelligence and cognitive ability, which suggests that people with lower levels of authoritarianism and conscientiousness, and higher levels of openness would perform better than people with higher levels of authoritarianism and conscientiousness and lower levels of openness (McCrae, 1994; Zeidner and Matthews, 2000; Moutafi et al., 2004; Deary et al., 2008; Stankov, 2009; Hodson and Busseri, 2012).

Consistent with findings in the 2013 OECD Survey of Adult Skills (Kuczera et al., 2016), we also observed that numeracy skills tend to decline with age amongst their voting cohort; that is, younger voters tended to perform better than older voters. We additionally note male participants outperformed female participants. While we had not previously considered or hypothesized these sex related differences, they are consistent with research observing that females appear to trail males in financial literacy (Fonseca et al., 2012; Mottola, 2013).

### Thinking styles

#### Cognitive reflection task

**Figure 4:**
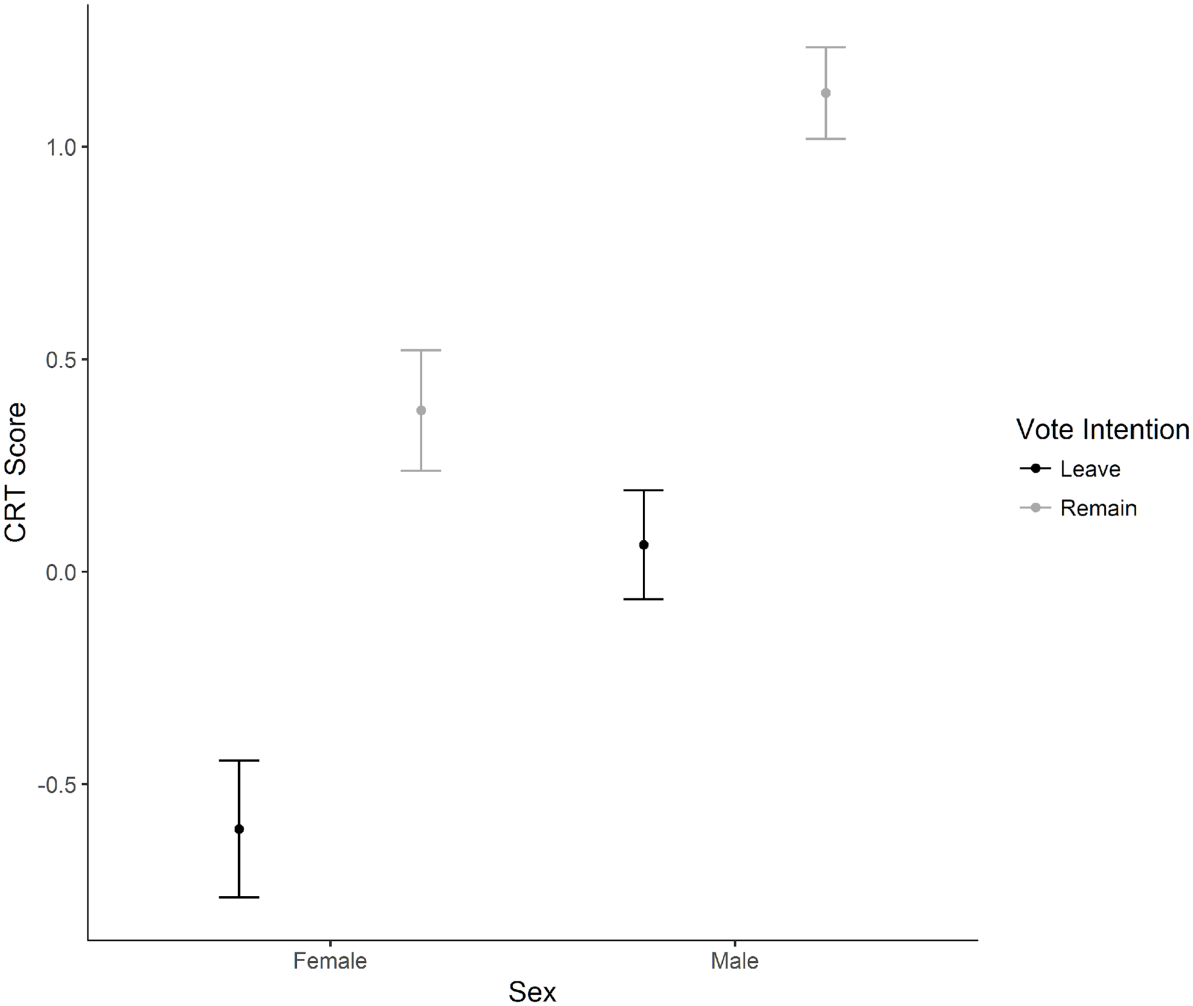
The interaction between sex and the cognitive reflection test score for vote intention. Error bars represent 95% confidence intervals of the mean.

A 2 (Sex) × 3 (Age) × 2 (Vote intention) between-subjects ANOVA was evaluated using the CRT score as the dependent variable. There was a main effect of vote intention, with Remain voters (*M* = 0.81, *SD* = 2.09) having higher scores than Leave voters (*M* = −0.19, *SD* = 2.11), *F*(1, 3,820) = 166.53, *p* < 0.001, η_p_^2^ = 0.04. An interaction of sex by vote intention was significant, *F*(1, 3,820) = 3.93, *p* = 0.05, η_p_^2^ < 0.01. Independent samples *t*-test with a Bonferroni correction were applied to male and female voters comparing vote intention. For females, Leave (*M* = −0.61, *SD* = 2.06) voters had lower scores than Remain voters (*M* = 0.38, *SD* = 2.20), *p* < 0.001, *d* = 0.46. The same pattern was found in males, with a slightly larger effect size where Leave voters (*M* = 0.06, *SD* = 2.10) had lower scores than Remain voters (*M* = 1.13, *SD* = 1.94), *p* < 0.001, *d* = 0.53. Figure 4 displays this interaction.

While cognitive reflection has been examined in relation to personality, authoritarianism, and conservatism, at the time of writing we were not aware of any research which had previously examined its relationship with attitudes to the EU. Nevertheless, our hypothesis was informed by the relationship between both openness and liberalism in relation to performance on the Cognitive Reflection Test. In finding that Leave voters rely more heavily on impulsive System 1 thinking than Remain voters, our results suggest that similar differences in thinking styles exist between Leave voters and Remain voters as they do between conservatives and liberals.

#### Wason card selection

For this analysis, we addressed the problems of homogeneity and heteroscedasticity by using a 2 (Sex) × 3 (Age Group) × 2 (Vote intention) permutation ANOVA. A significant main effect of vote intention emerged for the abstract reasoning cards, with Leave voters (*M* = 0.40, *SD* = 0.75) scoring lower than Remain voters (*M* = 0.68, *SD* = 0.91), *MSE* = 11.71, *p* < 0.001, iterations = 5,000, *d* = 0.32. This finding supports the hypothesis wherein Leave voters perform worse overall than Remain voters in an abstract reasoning setting.

**Figure 5:**
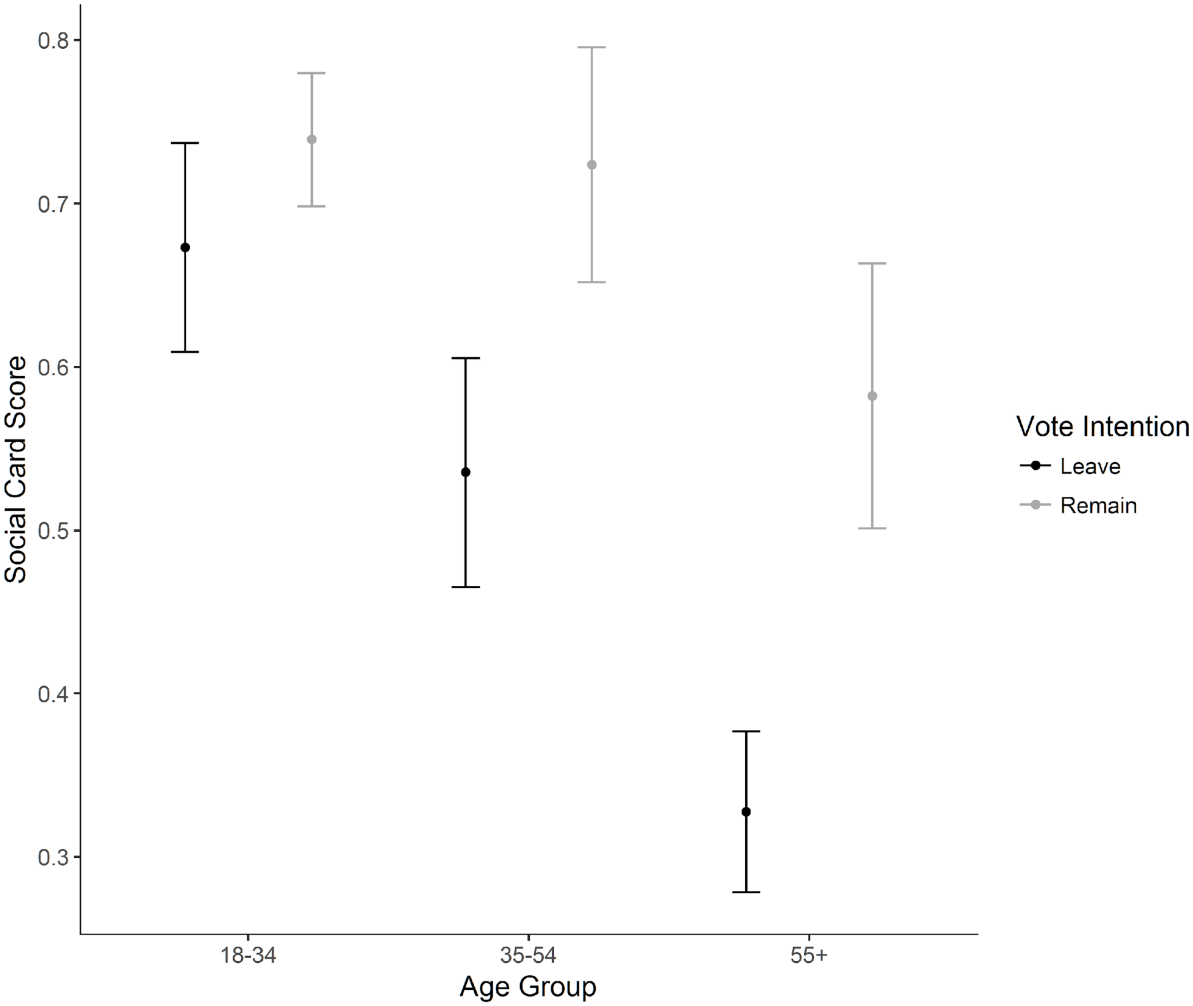
Mean scores for the social reasoning cards in the Wason card selection task for voting intention and age groups. Error bars represent 95% confidence intervals of the mean.

The same permutation ANOVA was analyzed for the Wason Card selection task focusing on social reasoning, where a significant interaction between age and vote intention was found, *MSE* = 0.74, *p* = 0.02, iterations = 5,000. For post-hoc analyses, pairwise permutation tests were run between different EU referendum voters, split on the variable of age group. For participants 18–34, there were no significant differences on card selection scores between Leave voters (*M* = 0.67, *SD* = 0.47) and Remain voters (*M* = 0.74, *SD* = 0.44), *p* = 0.08, *d* = −0.15. For participants 35–54 years of age, Leave voters (*M* = 0.54, *SD* = 0.50) had lower card selection scores than Remain voters (*M* = 0.72, *SD* = 0.45), *p* < 0.001, *d* = 0.39. For participants 55 and above, Leave voters (*M* = 0.33, *SD* = 0.47) had lower scores than Remain voters (*M* = 0.58, *SD* = 0.49), *p* < 0.001, *d* = 0.53. Figure 5 shows the social Wason card selection score for the interaction between age group and vote intention. This finding only partially supports our hypothesis, in that there was no significant difference was found between voters 18–34 years of age. However, Remain voters performed significantly better with participants 35 and older, which does not support our hypothesis.

As with cognitive reflection, we were not aware of any research which had previously examined whether a relationship exists between performance at the Wason selection task or deductive reasoning and attitudes to the EU. Further, we were unable to identify research which had examined performance on the Wason selection task in relation to political orientation, personality and attitudes to the EU. Therefore, in contrast to the other hypotheses in the present paper, these hypotheses were more speculative.

Remain voters’ performance advantage over Leave voters at the abstract reasoning task may, in part, be a reflection of their numeracy abilities. We subsequently identified prior research, which found that mathematics students performed better than history students (Inglis and Simpson, 2004). It is therefore plausible that numeracy or psychological constructs related to numeracy play an important role in abstract reasoning performance differences between Leave and Remain voters. It may also be possible that cognitive reflection plays a role, preventing participants from making intuitive but incorrect selections. There may be a simpler explanation, such as increased exposure to abstract reasoning problems. Nevertheless, these differences would benefit from further research.

Turning to the social rule, our hypothesis that performance differences may be reduced or eliminated were informed by the authoritarian characteristic of a need for order and the upkeep of societal norms. With the exception of the 18–34 age group, our results did not support this hypothesis. Future research should consider the effects of both numeracy and cognitive reflection in relation to performance at both the abstract and social rule selection tasks.

### Biases

#### Dunning-Kruger

**Figure 6:**
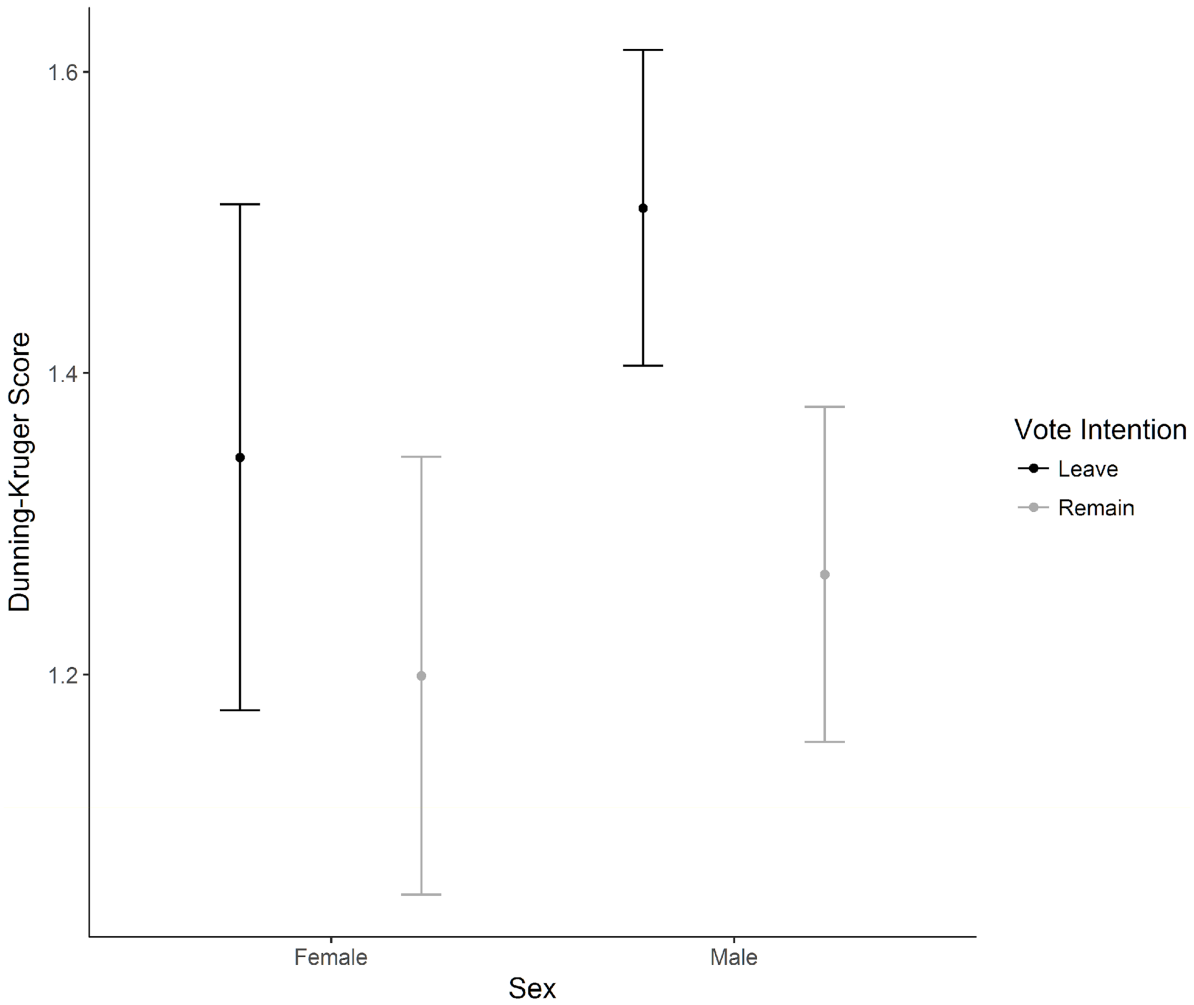
Means for the interaction between sex and vote intention with Dunning-Kruger bias scores (amount of overestimation) based on predicted Wason test scores minus the actual scores. Error bars represent 95% confidence intervals of the mean.

A 2 (Sex) × 3 (Age) × 2 (Vote intention) ANOVA was performed on the difference between correct guesses and predicted correct guesses, examining the Dunning-Kruger over or underconfidence bias effect. There was a suggestive interaction between vote intention and sex, *F*(1, 1,498) = 4.01, *p* = 0.045, η_p_^2^ < 0.01. To examine this interaction relevant to our hypothesis, differences between Leave and Remain voters were examined split on the variable of sex. Considering males, Leave voters (*M* = 1.51, *SD* = 1.21) had higher scores than Remain voters (*M* = 1.27, *SD* = 1.23), *p* < 0.01, *d* = 0.20, that is, male Leave voters were more susceptible to the Dunning-Kruger overconfidence bias effect than Remain voters. Considering females, there were no significant differences between Leave (*M* = 1.34, *SD* = 1.33) and Remain (*M* = 1.20, *SD* = 1.24) voters, *p* = 0.20. Figure 6 shows the interaction between sex and vote intention. These findings only partially support our hypothesis that both Leave and Remain voters would be similarly susceptible to the Dunning-Kruger bias effect, highlighting unexpected differences in susceptibility to the Dunning-Kruger effect in male voters.

There was a significant main effect of age, *F*(2, 1,498) = 31.37, *p* < 0.001, η_p_^2^ = 0.04. Pairwise *t*-tests using a Bonferroni correction were then analyzed. Participants 55 and older (*M* = 1.68, *SD* = 1.14) overestimated scores more than both participants aged 35–54 (*M* = 1.43, *SD* = 1.22, *p* < 0.01, *d* = 0.30) and 18–34 (*M* = 1.06, *SD* = 1.26, *p* < 0.001, *d* = 0.52). Participants 35–54 also overestimated scores more than participants (18–34, *p* < 0.001, *d* = 0.21).

At the time of writing, we were unaware of any studies which had explored the Dunning-Kruger over or underconfidence bias effect in relation to political orientation or personality, and we therefore felt that it would be reasonable to hypothesize that the effects of the bias would be broadly similar on both Leave and Remain voters. The results may be reflective of age-related differences between Leave and Remain voters, with younger voters being less susceptible to the Dunning-Kruger effect than older voters. However, it is also possible that personality and thinking styles play a role. Subsequent analysis of available literature has identified that openness is positively correlated with accuracy and confidence, but not overconfidence (Schaefer et al., 2004; Haran et al., 2013). In light of this research, a further possible explanation for the unexpected result of male Leave voters being more susceptible to the Dunning-Kruger effect than Remain voters is that the combination of Remain voters’ openness, deductive reasoning abilities and System 2 thinking, may play a role in causing them to be slightly more critical of their own performance. The role of neuroticism should also be explored as there may be a possibility that a form of ‘depressive realism’ factors in people’s evaluations of their own performance. Since this interaction was not present in female voters, it is additionally possible that the nature of the task (deductive reasoning) may have increased the participatory motivation among male participants. Further research is required to better understand the role of personality in relation to overconfidence and whether the nature of the task provides a differing motivation for males and females to be more self-critical.

#### Ideologically motivated reasoning

A 2 (Sex) × 3 (Age) × 2 (Vote intention) × 3 (Experimental Condition: Control, Leave Bias, Remain Bias) between-subjects ANOVA was analyzed with test bias scores as the dependent variable. The test bias score captured the level of agreement with the post-test question. Results revealed a significant interaction between vote intention and experimental condition, *F*(2, 1,411) = 13.21, *p* < 0.001, η_p_^2^ = 0.02.

**Figure 7:**
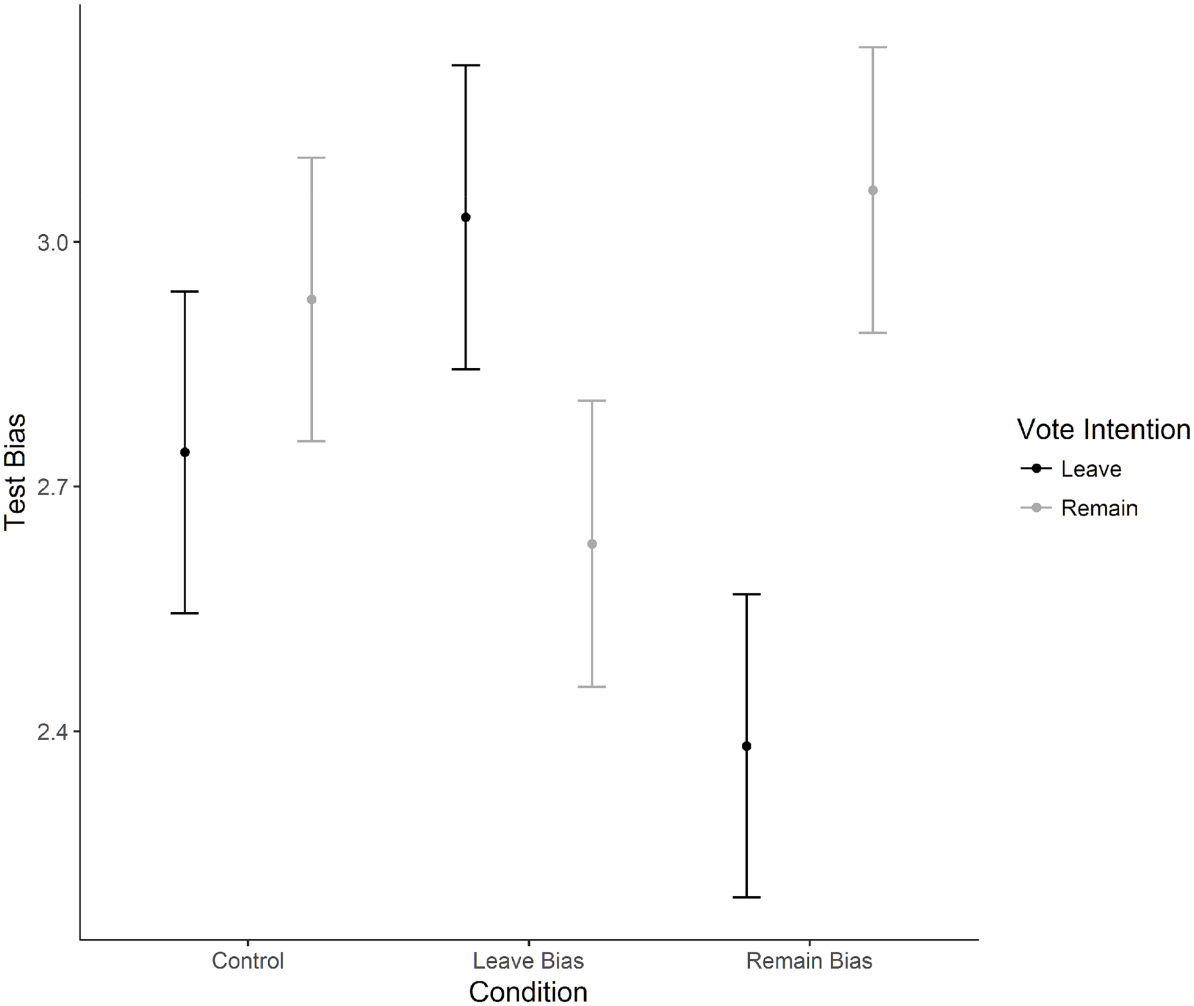
Means for test bias scores (level of agreement with post numeracy test statement) given experimental condition for ideological motivated reasoning. Error bars represent 95% confidence intervals of the mean.

For post-hoc analyses, pairwise *t*-tests with a Bonferroni correction were run between Remain and Leave voters, split on the experimental condition variable. For the control group, there was no significant difference between Leave (*M* = 2.74, *SD* = 1.47) and Remain (*M* = 2.92, *SD* = 1.41) voters, *p* = 0.160, *d* = 0.13. Considering the Leave biased condition, Leave voters (*M* = 3.03, *SD* = 1.43) reported higher levels of agreement than Remain voters (*M* = 2.63, *SD* = 1.48), *p* = 0.002, *d* = 0.27. Considering the Remain biased group, Leave voters (*M* = 2.38, *SD* = 1.40) reported lower levels of agreement than Remain voters (*M* = 3.06, *SD* = 1.41), *p* < 0.001, *d* = 0.48. Figure 7 shows the interaction between vote intention and condition on test bias.

In order to explore whether one group of voters were more influenced by their biases than the other group of voters, the experimental condition by vote intention interaction was explored further by examining the voter group levels of agreement for all three possible condition pairs (Control v Leave Bias, Control v Remain Bias and Leave Bias v Remain Bias) using independent t-tests with a Bonferroni correction.

Leave voters reported significantly lower agreement with the question “I think the word-problem test I just took supplies good evidence of how reflective and open-minded someone is” when they were told that “Remain voters tend to get more answers correct than Leave voters” (*M* = 2.38, *SD* = 1.40) as opposed to their agreement with the control condition when they were not provided with any further information (*M* = 2.74, *SD* = 1.47), *p* = 0.009, *d* = 0.25. The finding was reversed when Leave voters were told that “Leave voters tend to get more answers correct than Remain voters” (*M* = 3.03, *SD* = 1.43) as opposed to the control condition (*M* = 2.74, *SD* = 1.47), *p* = 0.037, *d* = 0.20. When comparing the levels of agreement of Leave voters between the two experimental conditions (Leave Bias v Remain Bias), the differences were significant, with a medium effect size, *p* < 0.001, *d* = 0.46.

Remain voters reported significantly lower agreement with the question “I think the word-problem test I just took supplies good evidence of how reflective and open-minded someone is” when they were told that “Leave voters tend to get more answers correct than Remain voters” (*M* = 2.63, *SD* = 1.47) as opposed to their agreement with the control condition where they were not provided with any further information (*M* = 2.93, *SD* = 1.41), *p* = 0.017, *d* = 0.21. No significant differences were observed in Remain voters’ agreement to the question “I think the word-problem test I just took supplies good evidence of how reflective and open-minded someone is” when they were told that “Remain voters tend to get more answers correct than Leave voters” (*M* = 3.06, *SD* = 1.41) as opposed to their agreement on the control condition where they were not provided with any further information, *p* = 0.284, *d* = 0.10. When comparing the levels of agreement of Remain voters between the two experimental conditions (Leave Bias v Remain Bias), the differences were significant, with a small to medium effect size, *p* < 0.001, *d* = 0.30.

Both voting groups were less likely to agree with statements which did not support the description intended to challenge their beliefs. Leave voters displayed greater differences between the two experimental positions and therefore appear to be more susceptible to ideologically motivated reasoning.

#### Ideologically motivated numeracy

This variable was scored wherein participants received one point for a correct answer and no points for an incorrect answer. Therefore, two binary logistic regressions were used to explore how sex, age, vote intention and condition (Condition: A and B, or C and D in separate regressions) would predict correct answers. Conditions A and B, the control conditions, required participants to interpret a contingency table with numbers reflecting the ability for skin cream to clear a rash; Conditions C and D, the experimental conditions, required participants to interpret a contingency table with numbers reflecting the effect of immigration on crime.

For the control questions, the overall model was significant, χ*^2^*(5) = 14.18, *p* = 0.014, Nagelkerke *R^2^* = 0.02; however, only the predictor of gender was significant, *b* = 0.42, *z* = 3.33, *p* = 0.001, indicating that men were more likely to correctly answer the test questions. Percent correct prediction for the incorrect group was 12.2%, while 90.4% of the correct group was accurately predicted (56.8% overall). For the experimental questions, the overall model was not significant, χ*^2^*(5) = 10.65, *p* = 0.059, Nagelkerke *R^2^* = 0.01, however, Remain voters were more likely to choose the correct answers, *b* = 0.33, *z* = 2.58, *p* = 0.010. Percent correct for the incorrect choices was 41.3 and 58.6% for the correct choices (51.5% overall).

**Figure 8:**
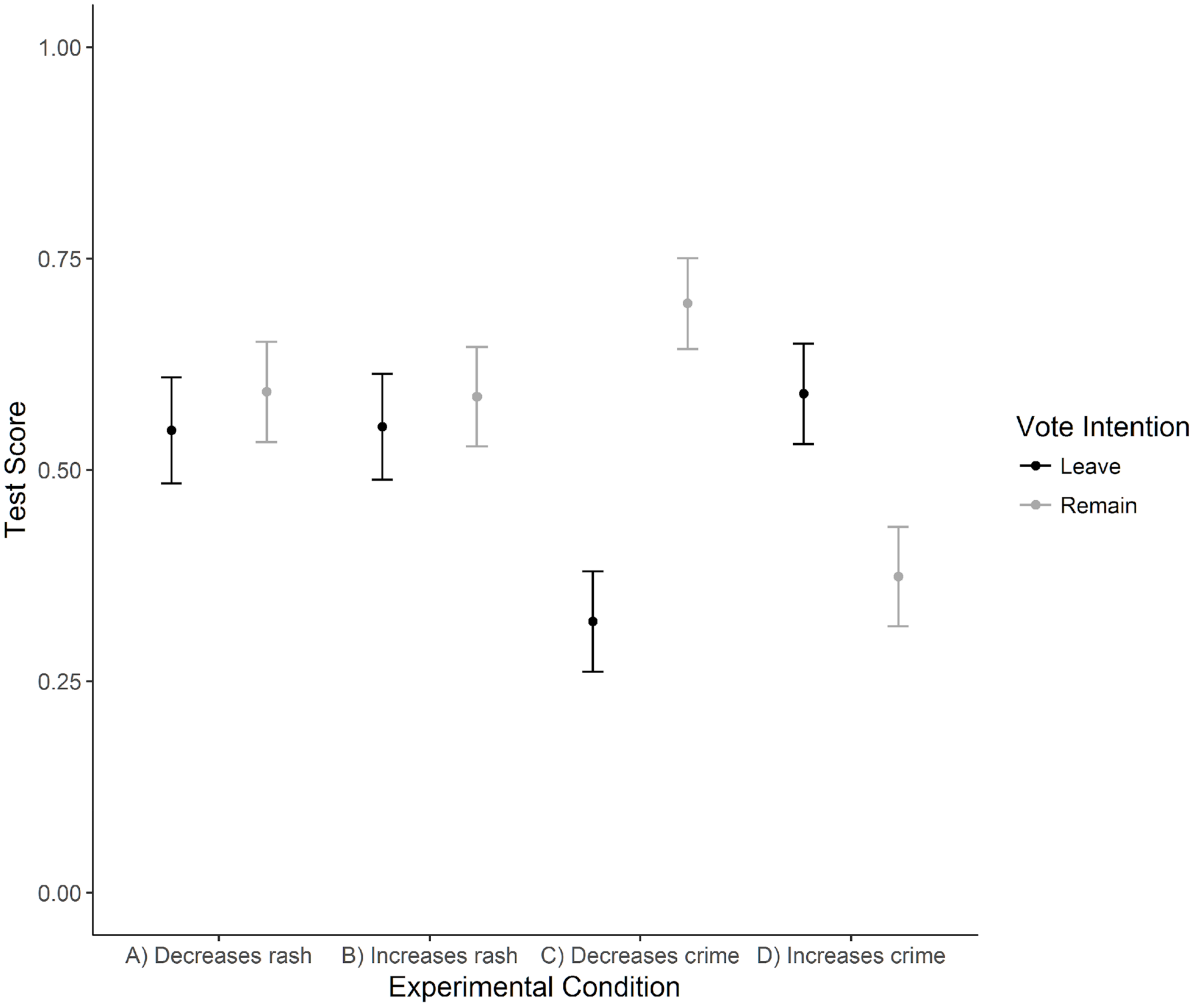
Scores for test condition and vote intention for test scores on the ideologically motivated reasoning task. Conditions A (Skin Cream Decreases Rash) and B (Skin Cream Increases Rash) were questions on the efficacy of skin cream, while conditions C (Immigration Decreases Crime) and D (Immigration Increases Crime) focused on immigration and crime.

In order to explore whether one group of voters appeared to be more influenced by their biases than the other group of voters, we examined the voter group performance for all six possible permutations of condition pairs (A v B, A v C, A v D, B v C, B v D and C v D) using 2 × 2 chi-square analyses. Figure 8 indicates the overall score for each voter group and condition combination. No significant differences were observed when examining Leave voters performance on the control conditions against their performance on the experimental condition that indicated that immigration increases crime (Condition A v D and B v D), χ*^2^*(1) =0.81, *p* = 0.369, *V* = 0.04 and χ*^2^*(1) = 0.65, *p* = 0.421, *V* = 0.04. When the experimental condition indicated that immigration decreases crime (Condition C), Leave voters showed a significant drop in performance against control conditions (A v C and B v C), χ*^2^*(1) = 24.31, *p* < 0.001, *V* = 0.22 and χ*^2^*(1) = 25.18, *p* < 0.001, *V* = 0.23, respectively. When comparing the task performance of Leave voters between the two experimental conditions (C v D), the differences were significant wherein Leave voters were more likely to score incorrectly on condition C, χ*^2^*(1) = 35.76, *p* < 0.001, *V* = 0.27.

In contrast to Leave voters, Remain voters showed a small, but significant improvement when their performance on the control conditions were compared with their performance on the experimental condition that indicated that immigration decreases crime (Condition A v C and B v C), χ*^2^*(1) = 6.12, *p* = 0.013, *V* = 0.11 and χ*^2^*(1) = 6.90, *p* = 0.009, *V* = 0.11. When the experimental condition indicated that immigration increases crime (Condition D), Remain voters showed a significant drop in performance against control conditions (A v D and B v D), χ*^2^*(1) = 24.30, *p* < 0.001, *V* = 0.21 and χ*^2^*(1) = 23.29, *p* < 0.001, *V* = 0.21, respectively. When comparing the task performance of Remain voters between the two experimental conditions (C v D), the differences were significant, χ*^2^*(1) = 56.22, *p* < 0.001, *V* = 0.32.

Both sides’ performance at interpreting the contingency table drops similarly when the results did not support outcomes intended to resonate with their beliefs. Supporting our hypothesis, Remain voters displayed a greater difference in performance between the two experimental positions and were therefore, arguably, more susceptible to ideologically motivated numeracy. Our findings are in line with Kahan (2013) who found that people with higher numerical literacy were more likely to engage in ideologically motivated numeracy. This may possibly explain the small differences between Leave and Remain voters; that is, as a population, Remain voters were found to have greater numerical literacy.

#### Framing

To analyze the Asian disease problem, a direct binary logistic regression analysis was conducted to investigate participant choice (*n*s = risk-avoidant: 1,549; risky: 1,987) predicted by age, sex, vote intention and which experimental condition the participant was exposed to (positive or negative framing). The full model, including age group, sex, vote intention, and positive or negative framing condition was significant, χ*^2^*(5) = 441.58, *p* < 0.001, Nagelkerke *R^2^* = 0.16. Overall, 66.77% of participants were correctly classified, with an improved classification for the risk-avoidant choice group (69.66%) over the risky choice group (64.52%). Participants in the negative framing condition were significantly more likely to choose the risky option, *b* = 1.44, *SE* = 0.07, *z* = 19.73, *p* < 0.001. Considering vote intention, there were no differences between Leave and Remain voters, *b* = − 0.01, *SE* = 0.07, *z* = −0.09, *p* = 0.93.

In order to explore whether one group of voters appeared to be more influenced by the framing effect than the other group of voters, the condition by vote intention interaction was explored further by examining the voter group choice (risk-avoidant v risky) in the positive frame against the voter group choice in the negative frame. Both Leave and Remain voters’ choices differed significantly between the frames, χ*^2^*(1) = 147.19, *p* < 0.001, *V* = 0.31 and χ*^2^*(1) = 259.11, *p* < 0.001, *V* = 0.36, respectively. The effect size was slightly higher for Remain voters.

A second direct binary logistic regression on the Sure gain/Sure loss problem was analyzed using participant choice (*n*s = risk-avoidant: 1,125, risky: 834) predicted again by age, sex, vote intention and positive or negative framing condition. The full model, including all predictors, was significant, χ*^2^*(5) = 317.76, *p* < 0.001, Nagelkerke *R^2^* = 0.20. 69.14% of participants were correctly classified overall. In this condition, there was improved classification for the risky choice group (72.18%) than with the risk-avoidant choice group (66.93%). As with the Asian disease problem, participants in the negative Sure gain/Sure loss framing condition were more likely to choose the risky option, *b* = 1.68, *SE* = 0.10, *z* = 16.65, *p* < 0.001. Contrary to the Asian disease problem, Remain voters were significantly more likely to choose the risk-avoidant option than Leave voters, *b* = −0.39, *SE* = 0.10, *z* = −3,83, *p* < 0.001. For post-hoc analyses, chi-square analyses were analyzed on Remain and Leave voters, split on the experimental condition variable. For the positive frame, there was no significant difference between Leave and Remain voters, χ*^2^*(1) = 2.86, *p* = 0.091, *V* = 0.05. Considering the negative frame, Remain voters were more likely to choose the risk-avoidant choice than Leave voters, χ*^2^*(1) = 13.57, *p* < 0.001, *V* = 0.12.

In order to explore whether one group of voters appeared to be more influenced by the framing effect than the other group of voters, the condition by vote intention interaction was explored further by examining the voter group choice (risk-avoidant v risky) in the positive frame against the voter group choice in the negative frame. Both Leave and Remain voters’ choices differed significantly between the frames, χ*^2^*(1) = 149.13, *p* < 0.001, *V* = 0.42 and χ*^2^*(1) = 145.02, *p* < 0.001, *V* = 0.36, respectively. In contrast to the differences observed in the Asian Disease problem, the effect size was higher for Leave voters.

The original hypothesis stating that there would be no significant differences between Leave and Remain voters was partially supported. In the Asian disease problem, there were no significant differences between Leave and Remain voters. However, when presented with the Sure gain/Sure loss problem, Leave voters appear to be influenced by the change in frame to a much greater extent than Remain voters. Leave voters were also more likely than Remain voters to choose the risky option for both positive and negative frames. This is not to say that Remain voters do not tend to choose the riskier options when presented with the negative Sure gain/Sure loss frame; they do, just to a lesser extent than Leave voters.

There are a number of possible explanations for the unexpected finding that Leave voters were less likely than Remain voters to choose the risk avoidant option in the negative Sure gain/Sure loss frame. The first possibility is that Leave voters were more willing to risk relatively small amounts of hypothetical money but not hypothetical lives. A second possibility is that the financial nature of the Sure gain/Sure loss problem motivated Leave voters to a lesser extent than it did Remain voters. A third possibility is that the Sure gain/Sure loss problem required a greater degree of numerical literacy than the Asian disease problem, which may therefore have given Remain voters a small advantage. In their original studies, Tversky and Kahneman (1981) observed clear differences in performance between the Asian disease problem and the Sure gain/Sure loss problem, with 78% of participants selecting the risky choice in the negatively framed Asian disease problem versus 87% of participants selecting the risky choice in the negatively framed Sure gain/Sure loss problem. It is possible that differences in numeracy, reasoning and personality play a role in the performance differences between Leave and Remain voters and should therefore be considered in future research.

## General discussion

In six separate studies, we found that Leave and Remain voters in the UK’s referendum on EU membership displayed statistically significant differences in personality, authoritarianism, numeracy and thinking styles. Voters on both sides of the referendum debate were susceptible to the cognitive biases tested, but often, unexpectedly, to different degrees. With respect to personality, authoritarianism and thinking styles, our results are consistent with the differences observed between liberals and conservatives spanning decades of research (Carney et al., 2008) and more recently between Europhiles and Eurosceptics (Schoen, 2007; Bakker and de Vreese, 2016). We found Remain voters to have higher levels of neuroticism and openness and lower levels of conscientiousness and authoritarianism than Leave voters. With regard to numeracy and thinking styles, we found that, when compared to Leave voters, Remain voters had higher levels of numerical risk literacy, were more likely to engage in analytical System 2 thinking, and tended to perform better in deductive reasoning tasks.

Turning to the studies which examined cognitive biases, we found that both Leave and Remain voters were susceptible to ideologically motivated reasoning, ideologically motivated numeracy, framing, and the Dunning-Kruger effect. Unexpectedly and depending on the bias being tested, this susceptibility often differed slightly between voting camps. First, while the results for the ideologically motivated reasoning and ideologically motivated numeracy studies showed that both Leave and Remain voters were affected to similar levels, we observed that Leave voters had a slightly greater susceptibility to ideologically motivated reasoning, while Remain voters had a slightly greater susceptibility to ideologically motivated numeracy. Second, the results from the framing studies showed that both Leave and Remain voters behaved similarly when presented with the Asian disease problem but displayed differences in the Sure gain/Sure loss problem. Specifically, in the negative frame of the Sure gain/Sure loss problem, we found that Remain voters were slightly less likely to pick the risky choice than Leave voters, although the effect was small and, overall, voters on both sides tended to pick the riskier choice in response to the negatively worded frame. Finally, as an overall population, both Leave and Remain voters were similarly susceptible to the Dunning-Kruger effect although male Leave voters were found to be more affected than male Remain voters. Although there was a great deal of similarity in voters’ susceptibility to the biases we tested, the small, but statistically significant, differences between voters warrants further investigation in future research.

Consistent with previous research observing age related changes in authoritarianism, conscientiousness, neuroticism and agreeableness (Cornelis et al., 2009; Soto et al., 2011), we found clear differences, with younger voters on both sides of the debate self-reporting lower levels of conscientiousness and authoritarianism, and higher levels of neuroticism than older participants intending to vote the same way. The picture for openness is a little more nuanced with extant research finding that openness tends to increase through adolescence, level off during adulthood, and decrease in old age (Roberts et al., 2006). We did not observe a decline in openness. Instead we found that openness did not change significantly with age for Leave voters but increased with age for Remain voters, with older Remain voters displaying significantly higher levels of openness than older Leave voters. Age related differences in openness, conscientiousness, and authoritarianism may provide further insight into the reason why older voters were more likely to vote to leave the EU than their younger counterparts. This nuance of age is an important area to better understand. It may be partially explained by research which has noted that decreasing levels of openness, together with an increasing ‘Need for Closure’ (NFC), in old aged voters appears to be associated with an age related rise in conservatism (Webster and Kruglanski, 1994; Roberts et al., 2006; Cornelis et al., 2009).

Turning to numeracy, deductive reasoning and cognitive reflection, we found that, in line with research into fluid intelligence (Tucker-Drob, 2011) and other cognitive abilities (Portugal Barcellos et al., 2016), younger voters outperformed older voters. With respect to cognitive biases, we found that younger voters were less susceptible to Dunning-Kruger and ideologically motivated reasoning than older voters. Age clearly plays a role in many of the factors explored in the present research and should therefore be further explored in future research. Furthermore, considering the observed declines in numeracy in the UK (Harding et al., 2012), future research should examine whether this age related difference might persist in future generations.

In these studies, we replicated and extended previous work to provide an insight into the psychological differences and similarities between Leave and Remain voters in the UK’s 2016 referendum on EU membership. Our findings provide additional context to extant referendum analysis. We have also opened up a number of overlapping areas for future exploration. Of these, we limit our discussion to narrow subset. In the subsequent paragraphs we arrange our discussion along two themes. First, we discuss how our findings could contribute to future research into regional differences and differences in levels of education between Leave and Remain voters. Pre- and post-referendum surveys have repeatedly highlighted regional and education differences between Leave and Remain voters. But it has yet to be explored whether personality, authoritarianism and cognition can help explain uncontrolled educational and regional effects. Secondly, we discuss our findings and their implications in relation to gaining a better understanding of the extent to which voters could be targeted and influenced through both traditional and social media, and also through their offline social networks.

Turning first to regional factors, the referendum results, together with pre- and post-referendum analysis have highlighted regional differences in voting patterns (Clarke and Whittaker, 2016; The Electoral Commission, 2016; Clarke et al., 2017). These differences can be further investigated in the context of the findings in the present studies and in the findings of research exploring regional differences in personality within the UK (Rentfrow et al., 2015). Specifically, when comparing electoral voting data (The Electoral Commission, 2016) with regional personality data (Rentfrow et al., 2015), the regions which voted in favor of leaving the EU were found to have higher T-Scores for conscientiousness (*M* = 51.92, *SD* = 8.70, *N* = 263) than regions which voted in favor of remaining in the EU (*M* = 45.69, *SD* = 11.34, *N* = 117). Remain voting regions, on the other hand, were found to have higher T-Scores for openness (*M* = 57.28, *SD* = 12.25, *N* = 117) than the regions which voted in favor of leaving the EU (*M* = 46.76, *SD* = 6.61, *N* = 263). These relationships are of particular interest as conscientiousness and openness are the personality traits most strongly correlated with authoritarianism within extant research (Sibley and Duckitt, 2008). However, further research is clearly needed in order to better understand the role that regional personality differences played in the referendum. For one thing, contrary to our findings that Remain voters had higher levels of neuroticism than Leave voters, Rentfrow et al. (2015) regional analysis found that Leave voting regions had higher T-Scores for neuroticism (*M* = 51.20, *SD* = 9.58, *N* = 263) than Remain voting regions (*M* = 47.24, *SD* = 10.42, *N* = 117).

Turning next to educational differences, pre- and post-referendum surveys highlighted that Leave voters tended to have lower levels of education than Remain voters (Clarke et al., 2017). On the surface, education appears to have been a factor in shaping voters’ attitudes to the EU, although the role of confounding variables such as numeracy, thinking styles, and openness have yet to be investigated. For instance, prior research has noted that “education tends to socialize students to have more tolerant, pro-outsider, views of the world” (Hainmueller and Hiscox, 2007, p: 405). However, upon further investigating the role of education on attitudes to immigration, Hainmueller and Hiscox (2007) found that once cultural values and economic literacy were taken into account, they accounted for roughly 65% of the uncontrolled effects of education on support for immigration. That is, Hainmueller and Hiscox (2007, p: 438) suggest that “education may be more of a symptom of the cultural divide between the two groups than it is a cause.” In light of the results in present paper, future research should therefore examine the roles of variables such as numeracy, intelligence, and openness; all of which are correlated with educational attainment (McCrae, 1994; Zeidner and Matthews, 2000; Furnham et al., 2005).

Additionally, Hainmueller and Hiscox (2007) found that college education has a greater influence on support for immigration than secondary education, supporting prior work with similar observations (Chandler and Tsai, 2001). Chandler and Tsai (2001) note that education fosters tolerance by increasing students’ knowledge of foreign cultures, raising levels of critical thinking and through exposures to a more diverse social network. Recent research has explored the effect of education on personality (Dahmann and Anger, 2014). However, at the time of writing, no studies had been identified which have examined whether personality influences students’ decisions in the first place to continue beyond secondary education. Considering the regional personality differences noted by Rentfrow et al. (2015), this may prove to be an important aspect to better understand. Future research should include an examination of the role of personality, numeracy, and thinking styles in the motivations for both applying to and attending University and other tertiary education institutes.

The study in the present paper and the study that it replicated (Kahan et al., 2017) focused on topics (immigration and gun control respectively) which have been found to split opinion along an authoritarian dividing line. The results from our examination of ideologically motivated numeracy showed that both Leave and Remain voters suffered a significant decline in performance when interpreting numbers contrary to their assumed ideological beliefs. Considering the differences in authoritarianism observed in the present research, it would appear that authoritarianism played a significant part in the ideological motivation behind Leave and Remain voters. We are not, however, suggesting that authoritarianism was solely responsible for the way in which people voted. There are many reasons contributing to voters’ choices. However, of interest, given the nature of the campaigns, is prior research which has highlighted how factors such as fear and uncertainty can influence a voter’s choice (Abramson et al., 2007; Vasilopoulos et al., 2018).

Extant research has observed that when policy choices involve topics such as immigration and terrorism, which tend split opinion along an authoritarian schism, there is an interaction between authoritarianism and the way in which people perceive a threat (Hetherington and Weiler, 2009; Hetherington and Suhay, 2011). That is, while people with higher levels of authoritarianism are already likely to support authoritarian policies, people with lower levels of authoritarianism become increasingly likely to support authoritarian policies the greater they perceive a threat to be. Subsequent research has additionally identified that the primary emotions of fear and anger play a significant role in influencing support for authoritarian policies (Vasilopoulos et al., 2018).

Considering the role of threat perception introduced in the previous paragraph, it is possible that the same or similar emotions factored in the referendum. This is because issues such as immigration and sovereignty were salient topics; and topics which have all been shown to divide opinion along an authoritarianism fissure (Sales, 1972; Lavine et al., 1999; Hetherington and Weiler, 2009). For example, it may have been possible to influence voters with low authoritarianism, who might otherwise have voted Remain, to vote Leave by increasing their concerns and therefore fears about immigration. Conversely, and additionally considering neuroticism, it may also have been possible to influence voters with high authoritarianism, who might otherwise have voted Leave, to vote Remain by increasing their concerns and therefore fear about economic risks. Future research should therefore explore the role of perceived threats in relation to each of the key referendum topics, with a focus on emotions associated with fear and anger. Given allegations of the use of fear tactics and misinformation leveled at both campaigns and post-referendum analysis of perceived risks (LeDuc, 2016; Clarke et al., 2017), this topic is an important area of future research. A successful exploitation of these interactions could have the potential to significantly alter political outcomes, especially in referendums and elections where victories are secured by a small number of percentage points.

The findings in the present paper and subsequent discussion highlight a number of social and political implications. Of these, we have limited our discussion to a subset that we considered to be especially important given the backdrop of allegations of psychographic targeting and marketing in online social networks, selective reporting, misinformation, and media bias. While the use of messages designed to resonate with certain personality traits and not others are not new, nascent research suggests that data from online social networks could enable anyone to create and amplify messages which play on inherent beliefs and primary emotions. These messages can then be directed to increasingly narrowly defined audiences (Matz et al., 2015, 2017; Sumner, 2017). While much of the recent media attention has focused on advertising, online social networks could be leveraged in other ways, such as testing and refining messages for use in wider campaigns, both on and offline. Future research should therefore seek to better understand how voters are targeted, how messages are spread and amplified, and how voters process unfiltered content and a volume of content that is increasingly difficult to fact check, especially given the Cambridge Analytica scandal that was discovered after the data collection for this manuscript (Facebook–Cambridge Analytica data scandal, 2022). Attempts should also be made to determine whether and how voters could be educated about their biases in order to reduce their possible future exploitation. Furthermore, the findings presented in the present paper raise important questions regarding the use of direct democratic mechanisms such as referendums and especially binary, winner-takes-all referendums in respect of ideologically charged choices.

### Limitations

The research methods employed in the present study are subject to a number of limitations. The first limitation is that the surveys were all self-selection, which may therefore have introduced a selection bias. However, through an inspection of the descriptive statistics, our sample appears to be reasonably consistent with voting intentions across age and sex groups reflected in official polls (The Electoral Commission, 2016). The second limitation is that the research was based solely on self-report questionnaires, which participants could manipulate, producing a measurement error (Couper, 2000). Self-reporting, however, is a widely used method and with such a large sample size we consider it unlikely that the results would be significantly skewed by individuals wishing to manipulate their scores. Further, we took steps to prevent participants from resubmitting their survey results (Jouravlev, 2004) and added time stamps in order to detect when participants may have been rapidly clicking through the form. In order to address the limitations inherent in self-report studies, future research could additionally make use of interview led data collection.

A limitation of the analyses presented may be that voting presented is simply a proxy for political orientation, wherein these results would be consistent with previous research on personality, authoritarianism, and political ideology which leads to the match in voting behavior (Johnston and Wronski, 2015; Johnston, 2018). An additional limitation of the study may be the sampling procedure, as participants were solicited through Facebook ads. Participants were recruited, and provided survey responses, prior to the referendum. At that time, there was scant public knowledge that Cambridge Analytics were involved in the alleged collection of Facebook user information for use in political campaigns; therefore, we do not consider that knowledge of Cambridge Analytics influenced study participation (Facebook–Cambridge Analytica data scandal, 2022). Despite a relative lack of public knowledge of Cambridge Analytica and the practice of social media-based voter profiling and targeting, it is possible that voters had been influenced by online messaging and targeting by the time they participated in this research. The final limitation is that the present study was based on six separate surveys made available at different stages of the referendum campaigns. It is therefore possible that some attitudes may have shifted during the referendum campaigns. Future research could consider giving each participant all of the studies to complete and also consider a longitudinal study which observes how and indeed, whether, attitudes shift during a referendum.

## Conclusion

We found that Leave voters had higher levels of authoritarianism and conscientiousness than Remain voters. We also found that Leave voters were less numerate and more likely to engage in impulsive, System 1 thinking than Remain voters. Remain voters on the other hand self-reported higher levels of neuroticism and openness and appeared to be slightly more risk-averse than Leave voters. The results also illustrate that voters on both sides of the debate were similarly susceptible to cognitive biases. Younger voters were more numerate, had greater deductive reasoning ability, and were generally less susceptible to cognitive biases than older voters. In the context of the referendum, it is a plausible conjecture that a significant portion of voters aligned their choices based on pre-existing beliefs which appear to have been primarily structured by authoritarianism. Working in concert, cognitive biases are likely to have reduced voters’ ability to accurately process evidence which did not support pre-existing beliefs, further accentuating differences in opinion. The results from these studies add to ongoing research into the societal schisms in the UK. They suggest that future political debate could be strongly influenced by topics on which voters are divided according to differing levels of authoritarianism. Additionally, the present study contributes to research seeking to better understand the extent to which knowledge of voters’ core psychological characteristics and biases could be exploited in order to influence the way in which those voters form early opinions and subsequently process and interpret information. The importance of such research has been underlined in nascent studies which have demonstrated the effectiveness of categorizing, targeting, and influencing online social network users based on their personalities (Matz et al., 2015, 2017; Sumner, 2017).

The research presented in the present manuscript raises important questions regarding the use and framing of numerical and non-numerical data during UK political campaigns. In a situation where “In general, political campaign material in the UK is not regulated, and it is a matter for voters to decide on the basis of such material whether they consider it accurate or not” (The Electoral Commission, 2018) the research also raises the question of whether existing regulatory controls need to be amended. Not only do many voters lack the skills to critically evaluate the information, which is being presented, their inherent beliefs and biases clearly influence the way in which they process this information. Considering these factors, a fundamental question is raised as to whether direct democracy in the form of binary, winner-takes-all, referendums is an appropriate mechanism for deciding major and complicated political issues, such as constitutional changes. More broadly, constitutions may need to be adapted to take into account fundamental shifts in societies’ use of technology and consumption of information.

## Data availability statement

Data information can be found at https://osf.io/urt63/.

## Ethics statement

Ethical approval was received from the Online Privacy Foundation Ethical Board in London, United Kingdom (2016/12–27/2). The patients/participants provided their written informed consent to participate in this study.

## Author contributions

CS drafted the original version of this manuscript, designed the study, and collected the data. JS and EB analyzed the data, wrote the results, and created the visualizations. All authors reviewed and commented on this manuscript. All authors contributed to the article and approved the submitted version.

## Conflict of interest

The authors declare that the research was conducted in the absence of any commercial or financial relationships that could be construed as a potential conflict of interest.

## Publisher’s note

All claims expressed in this article are solely those of the authors and do not necessarily represent those of their affiliated organizations, or those of the publisher, the editors and the reviewers. Any product that may be evaluated in this article, or claim that may be made by its manufacturer, is not guaranteed or endorsed by the publisher.

## Footnotes

^1^ https://osf.io/urt63/

^2^Both of these tasks can be viewed with an explanation at https://www.psychologyinaction.org/2012-10-07-classic-psychology-experiments-wason-selection-task-part-i/

^3^To learn more about permutation tests, see https://youtu.be/AcQqnwg6qsw
